# Changes in the Physicochemical and Bioactive Properties of Yerba Mate Depending on the Brewing Conditions

**DOI:** 10.3390/molecules29112590

**Published:** 2024-05-31

**Authors:** Katarzyna Najman, Rafał Rajewski, Anna Sadowska, Ewelina Hallmann, Krzysztof Buczak

**Affiliations:** 1 Department of Functional and Organic Food, Institute of Human Nutrition Sciences, Warsaw University of Life Sciences, Nowoursynowska 159c, 02-776 Warsaw, Polandanna_sadowska@sggw.edu.pl (A.S.);; 2 Bioeconomy Research Institute, Agriculture Academy, Vytautas Magnus University, Donelaicio 58, 44248 Kaunas, Lithuania; 3 Department of Surgery, Faculty of Veterinary Medicine, Wroclaw University of Environmental and Life Science, Pl. Grunwadzki 51, 50-366 Wroclaw, Poland

**Keywords:** yerba mate, Paraguayan holly (*Ilex paraguariensis* A. St.-Hil.), single and double brewing, color parameters L*a*b*, tannins, caffeine, carotenoids, polyphenols, antioxidant activity

## Abstract

Yerba Mate drink made from dried and crushed leaves and twigs of Paraguayan holly (*Ilex paraguariensis* A. St.-Hil.), which is a valuable source of bioactive substances, in particular antioxidants. The available literature lacks data on changes in the content and profile of bioactive compounds such as tannins, caffeine, the phenolic acid profile of flavonoids and carotenoids, as well as total polyphenol content and antioxidant activity in Yerba Mate infusions depending on different brewing conditions, and how different brewing conditions affect the physicochemical properties of these infusions. Therefore, this study evaluated the physicochemical properties of dried and Yerba Mate infusions prepared via single and double brewing processes at 70 °C and 100 °C. The organoleptic evaluation, as well as the instrumental color measurement, showed significant changes in the total color difference (Δ*E*) and the L*a*b* chromatic coordinates of dried Yerba Mate samples and their infusions. Moreover, the research showed higher contents of tannins (mean 1.36 ± 0.14 g/100 g d.m.), caffeine (mean 17.79 ± 3.49 mg/g d.m.), carotenoids (mean 12.90 ± 0.44 μg/g d.m.), phenolic acids (mean 69.97 ± 7.10 mg/g d.m.), flavonoids (mean 5.47 ± 1.78 mg/g d.m.), total polyphenols (mean 55.26 ± 8.51 mg GAE/g d.m.), and antioxidant activity (mean 2031.98 ± 146.47 μM TEAC/g d.m.) in single-brewed Yerba Mate infusions compared to double-brewed (0.77 ± 0.12 g/100 g d.m., 14.28 ± 5.80 mg/g d.m., 12.67 ± 0.62 μg/g d.m., 57.75 ± 8.73 mg/g d.m., 3.64 ± 0.76 mg/g d.m., 33.44 ± 6.48 mg GAE/g d.m. and 1683.09 ± 155.34 μM TEAC/g d.m., respectively). In addition, infusions prepared at a lower temperature (70 °C) were characterized by a higher content of total polyphenols and higher antioxidant activity, in contrast to the tannin and carotenoid contents, the levels of which were higher at 100 °C than at 70 °C. Considering the high amount of bioactive ingredients, in particular antioxidants, and a wide range of health benefits, it is worth including Yerba Mate in the daily diet.

## 1. Introduction

Yerba Mate is a drink made from the dried and fragmented leaves and twigs of Paraguayan holly (*Ilex paraguariensis* A. St.-Hil.), a plant belonging to the holly family (*Aquifoliaceae*), the holly genus (*Ilex*), which includes over 450 species, such as *Ilex brevicuspis*, *Ilex dumosa*, *Ilex integerrima*, *Ilex affinis*, occurring in South America [1,2,3].

Yerba Mate is the national drink of Brazil, Argentina, Uruguay, and Paraguay [3,4], and its consumption is deeply rooted in the tradition of these countries, referring to the old customs of the Guarani Indians, who treated this drink as a symbol of reconciliation and friendship [1,2,5]. Yerba Mate is closely related to the culture and customs of South American countries [2,4], but in recent years its consumption outside the home continent, mainly in Europe [4,6], in particular Poland [7], has been rapidly increasing.

The beneficial effects of Yerba Mate consumption on health were already appreciated by its discoverers [2]. Nowadays, the literature describes Yerba Mate as a valuable source of biologically active substances supporting the body functioning and playing an important role in the prevention as well as in the treatment of various diseases, including lifestyle diseases [8]. The pro-health properties of Yerba Mate, widely described in the literature may contribute to the prevention of diseases such as obesity [8], insulin resistance, or diabetes [9], e.g., by reducing body weight and the amount of food consumed, increasing the rate of gastric emptying, lowering insulin level, and improving glucose and lipid metabolism (closely related to adipose tissue), as well as increasing the concentration of glucagon-like peptide 1 (GLP-1) and leptin, factors responsible for the feeling of satiety [8,9]. Many studies confirm also the important role of Yerba Mate in the prevention of cardiovascular diseases [6,10]. Yerba Mate may also have anti-mutagenic, anti-proliferative, cytotoxic, and anti-cancer effects in relation to various cancers [9,11,12]. The beneficial effect of Yerba Mate in the course of neurological and neurodegenerative diseases is also known [13]. The flavones, caffeine, theobromine, and theophylline present in this plant and in its infusions have a neuroprotective effect, improve mood, cognitive functions, concentration, and stimulate and support mental work, as well as affecting the increase in the number of synapses and extend the life of nerve cells [13,14].

Paraguayan holly (*Ilex paraguariensis* A. St.-Hil.) is a plant characterized by a high content of bioactive ingredients such as purine and xanthine alkaloids, polyphenols, and other antioxidants or tannins that have a positive effect on human health [4,8,9,15,16,17,18,19].

The content of purine alkaloids, mainly including caffeine, also present in coffee, tea or cocoa [13], in Yerba Mate is from 41.82 to 82.4 mg of caffeine per 150 mL, which, in terms of the content of this ingredient, puts it on par with green tea (from 11.83 to 51.57 mg/150 mL) or coffee (from 42.27 up to 120.89 mg/150 mL) [14].

Yerba Mate has strong antioxidant properties, resulting from a high content of carotenoid and phenolic components [4,8,9,15,16,17,20,21,22,23]. Common antioxidants in Yerba Mate are phenolic acids such as chlorogenic, caffeic, feluric, and gallic acids [24]. Other phenolic components with antioxidant activity are flavonoids, among which rutin, quercetin 3-rhamnoside and 3-glucoside, kaempferol 3-rhamnoside and 3-glucoside, and luteolin diglycoside are the most common in Yerba Mate [25,26,27].

A very rarely described group of phenolic components in Yerba Mate are tannins [18,19]. These include compounds with a molecular weight from 500 to even 3000 Da and a large number of functional groups, thanks to which they bind to proteins, polysaccharides [28,29], metal ions, or alkaloids, forming water-insoluble complexes [30,31].

Many beneficial properties are attributed to tannins, including astringent and alkaloid-binding properties (helpful in the treatment of diarrhea and food poisoning) [29,32,33] or antifungal and antiviral properties (thanks to binding of the microorganisms’ cell wall polysaccharides and proteins) [32,33,34]. The ability to complex metals inhibits, e.g., the activity of hyaluronidase, lipoxygenase, protein kinase C, or ACE (angiotensin converting enzyme), which is why tannins also have anti-inflammatory properties [35]. The antidiabetic properties of tannins are known from their ability to slow down glucose absorption and lower blood sugar levels [36]. Tannins shape the appropriate astringent, sour, or bitter taste characteristics of wine or tea [37].

Due to the richness of ingredients and their high content and biological activity, Yerba Mate is increasingly used as an ingredient of functional food, which is confirmed by the appearance of products with Yerba Mate additions on the market, such as pasta, bakery and pastry products, ice cream, smoothies and shakes, lemonades, dessert coffees, alcoholic cocktails, and others [38]. Due to the high caffeine content, it is also used in the production of energy drinks and various types of cold drinks [14]; however, both in South America and Europe, Yerba Mate is most often consumed as a traditional brew [2,4,5,16].

The optimum brewing temperature for Yerba Mate is 65–80 °C, while, according to the traditional brewing process, 75% of the vessel should be occupied by dried material, in which case the brew has a heavy, bitter, and astringent taste and a dark, green, sometimes green-brown color [4,5]. Also, the mixtures of Yerba Mate products on the market differ in both the type and composition of the dried material, including the proportions of dried leaves to twigs and sticks [1,39,40]. Generally, there are two basic types of Yerba Mate, i.e., “elaborada” (or “elaborada con palo”) containing up to 30% twigs and sticks, and “despalada” (or “despalada sin palo”), containing up to 10% of these elements [40,41]. In practice, commercial products differ in the content of leaves, twigs, sticks, or dust; they may also contain the addition of dried herbs, fruits, or flowers [2,40], which makes Yerba Mate market products extremely diverse, both in terms of organoleptic and bioactive characteristics [26,40].

The contents of bioactive ingredients, as well as the biological activity and health-promoting properties of Yerba Mate, depend on many factors, including the origin of the Paraguayan holly [23], plant growth conditions, i.e., substrate, soil type, climate, degree of insolation or cultivation system [39,42], plant variety and morphotype [17,43], different parts of the plant [44], or leaf age [20,45]. Differences in the content of ingredients and bioactive properties also result from the composition and degree of fragmentation of commercially available mixtures [6,25,26,27,44,46,47], as well as the production technology and degree of processing of the Yerba Mate [3,16,20,21,42,45,46,48]. The key factors for the organoleptic and bioactive properties of Yerba Mate infusions are also the brewing methods and conditions, including temperature, time, and the number of infusions [16,45,48,49].

Similarly to tea, Yerba Mate is consumed in the form of a warm drink, an infusion of dried plant leaves, but these drinks are not the same. Tea is an infusion of Chinese tea leaves (*Camellia sinensis*), a plant originating in Asia, while Yerba Mate is an infusion of Paraguayan holly (*Ilex paraguariensis*), originating in South American countries [50]. In addition, tea is usually brewed in boiling or slightly cooled water, while for Yerba Mate brewing the water temperature is usually between 65 °C and 80 °C [2]. Most often, the process of brewing Yerba Mate in optimal temperature conditions lasts from 5 to 10 min, and, unlike tea, Yerba Mate is brewed many times, depending on consumer preferences [16].

From the consumer’s point of view, not only the color, aroma and taste are important, but also the content of ingredients determining the bioactive properties of Yerba Mate infusions [51,52,53]. While the health-promoting properties of Yerba Mate are well known and described in the literature, there are no reports on the organoleptic and physicochemical properties of infusions prepared in different conditions.

There is also a lack of data on changes in the content and bioactive compounds profile in Yerba Mate infusions, such as tannins, caffeine, and the phenolic acid profile of flavonoids and carotenoids, as well as total polyphenol content and antioxidant activity depending on different brewing conditions. Therefore, this study examined the influence of the temperature optimal for brewing Yerba Mate (i.e., 70 °C) and optimal for brewing most teas (i.e., 100 °C) as well as the number of brewing times (single and double) of Yerba Mate on the organoleptic characteristics (in particular color), physicochemical properties (ph, °Brix, osmolality), the content of bioactive ingredients, including tannins and caffeine, the profile of phenolic acids, flavonoids, carotenoids, and the content of total polyphenols and the antioxidant activity of the infusions obtained in this way.

## 2. Results and Discussion

### 2.1. Physicochemical Properties of Dried Yerba Mate and Its Different Infusions

**Figure 1:**
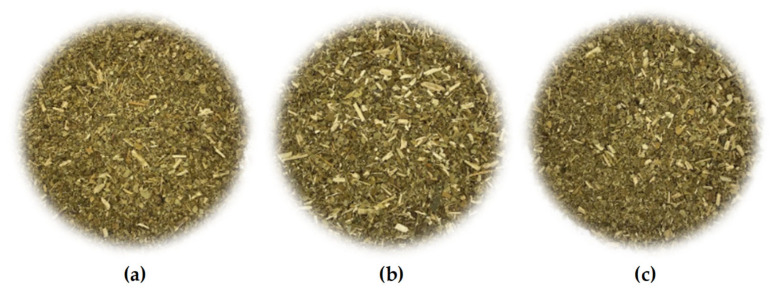
General appearance of dried Yerba Mate samples: YM-A (**a**), YM-B (**b**), YM-C (**c**).

The general appearance of dried Yerba Mate samples are shown in Figure 1. In the organoleptic assessment, all the tested Yerba Mate samples were loose and dry, but they differed in the degree of fragmentation and the structure of the mixture, resulting from the share of leaves, twigs and dust. The highest degree of fragmentation was found in YM-A (Figure 1a), with a small, but clearly visible, amount of fragmented twigs. This sample was also characterized by the highest dust content. The YM-C sample (Figure 1c) was characterized by a lower degree of decomposition of the mixture, a moderate content of dust and an average content of visible fragments of twigs with a large predominance of quite finely ground leaves. The sample with the lowest degree of fragmentation and the smallest dustiness, with fragments of finely cut twigs and sticks clearly visible and present in a relatively large amount in the mixture was the YM-B sample (Figure 1b).

All dried Yerba Mate samples had a strong, sweet, grassy, fresh hay-like aroma, with noticeable smoky notes. The most intense aroma was found in the YB-A sample, while the least aromatic was in the Yerba Mate YM-B sample.

In the organoleptic evaluation, the dried samples were characterized by a similar, green-brown, quite heterogeneous color, resulting from the varying degree of fragmentation of the mixture, and thus from the varying proportion of fragments of brightly colored twigs and sticks or dust. Samples with a greater share of twig fragments brightening of the mixture, were optically brighter. The most optically homogeneous composition of the mixture was distinguished by YM-A (with the highest degree of fragmentation), while YM-B was characterized by the least uniform color and its shade. No lumps, solid impurities or pests were found in any of the tested died samples. Foreign, non-specific aromas were also imperceptible, which proved the correct conditions of transport and storage.

One of the most important attributes of the appearance of food products, strongly affecting consumer acceptance, is color. Undesirable colors may induce the consumer to reject such products [54,55]. This attribute is the basic selection criterion for tea consumers [51]. In the case of Yerba Mate, color preferences depend on the country, with consumers in Brazil preferring light green, while in Argentina, Paraguay and Uruguay light olive, obtained as a result of the chlorophyll degradation during the Yerba Mate production [52].

From the point of view of the organoleptic assessment and the overall quality of products, it is important to determine the color of dried Yerba Mate and the effect of various brewing conditions of Yerba Mate on the color parameters of the infusions.

Table 1 presents the basic physicochemical parameters, such as moisture content and water activity (*a_w_*), as well as chromatic coordinates in the CIE LAB color space (L*a*b*) and browning index (BI) of dried Yerba Mate samples.

Dried Yerba Mate products were characterized by low water activity (average 0.38 ± 0.01) and low moisture content (average 5.50 ± 0.36%). Considering that the optimal *a_w_* for the development of most microorganisms is higher than 0.60 [56], low *a_w_* in the Yerba Mate samples proved the high durability and microbiological safety of these products, as well as the correct production, transport or storage process [19]. In the available literature, there is little data on water activity and moisture content in dried Yerba Mate, however, Frizon and Nisgoski (2020) [19] showed a similar moisture content in Yerba Mate products, ranging from 5.05 ± 0.8% (organic) to 5.21 ± 0.4% and even 5.94 ± 0.3% (conventional). These authors showed that at various stages of Yerba Mate production, the moisture content in green leaves from organic (65.27 ± 2.0%) and conventional (60.62 ± 1.7%) cultivation was reduced (by approx. 92%), also determining the color changes in dried products [19].

As the research shows, the dried Yerba Mate samples (Table 1) differed *(p* ≤ 0.05) in color L*a*b* parameters. The L* parameter, defining the lightness, ranged from 37.63 ± 1.63 to 74.00 ± 3.31, with the darkest YM-B, the brighter YM-A, and YM-C the brightest.

All dried Yerba Mate samples showed negative values of the a* parameter, which meant that they were characterized by a color shift towards shades of green, with the highest intensity of this color noted in the case of YM-C (−2.25 ± 0.38), significantly (*p* ≤ 0.05) lower for YM-B and YM-A.

The tested droughts were also characterized by clear shades of yellow color, reaching positive values of the b* parameter (defining yellow tones), which on average for all analyzed Yerba Mate samples amounted to 31.78 ± 3.71. The highest values for this parameter were found in samples YM-A and YM-C (average 33.69 ± 2.93), which meant that their color was mostly shifted towards yellow shades, unlike YM-B, for which the value of parameter b* were significantly (*p* ≤ 0.05) the lowest (27.95 ± 1.30).

In the literature, there are few studies on the color parameters of Yerba Mate. Frizon and Nisgoski (2020) [19] showed that the color of fresh holly leaves varied depending on the growing conditions and changed during the different steps of Yerba Mate production. Green leaves were characterized by negative (−*a*) values in the range from −8.50 ± 0.69 to 4.48 ± 1.16, after bleaching (2–3 min burning at 500–600 °C) the values of the a* component ranged from −7.96 ± 0.66 to −6.04 ± 1.31 (organic and conventional cultivation, respectively). On the other hand, the drying and grinding processes gradually led to the loss of green color, reaching values from −3.92 ± 1.74 to 3.49 ± 0.65, and after storage from −2.03 ± 0.19 to 0.12 ± 0.47 [19], which was close to the values in this study. A similar trend was found by Schmalko and Alzamora (2001) [47], although their values for the coordinate (−*a*) were much lower (from 9.13 in fresh to −5.46 in dried Yerba Mate leaves).

As a result of heat treatment, in addition to the loss of green color (−a*), the intensity of yellow color (b*) and lightness (L*) also increase [19]. In the studies of Frizon and Nisgoski (2020) [19], in the industrial Yerba Mate production, dried samples reached values for the chromatic coordinate b* from 17.87 ± 1.87 to 31.59 ± 3.77, and therefore similar to those in this study (Table 1), lower values for parameter b* (from 17 to 21) was found by Schmalko and Alzamora (2001) [47]. These differences can be explained by age of leaves, varied content of chlorophyll a and b in fresh leaves and the rate of degradation of these pigments depending on the time and temperature of leaf drying [19,47], as well as the degree of fragmentation and the rate of tissue dehydration during the Yerba Mate production [47]. Differences in color, especially in the brightness of dried Yerba Mate, also depend on the growing conditions (conventional vs. organic), storage time, or moisture content [19].

According to the literature, higher moisture content is accompanied by a decrease in the value of L* and b* color parameters [19], which was confirmed by the research conducted in this study (Table 1). The YM-C with the significantly (*p* ≤ 0.05) lowest moisture content (5.05 ± 0.02%) was characterized by the significantly highest value of the L* parameter (74.00 ± 3.31) and the lowest browning index (52.91 ± 2.28), and therefore the highest brightness. In turn, the YM-B sample, with significantly (*p* ≤ 0.05) the highest moisture content (5.86 ± 0.04%), was characterized by the lowest value of the L* parameter (37.63 ± 1.63) and was the darkest, displaying the highest browning index (115.64 ± 2.12).

Yerba Mate is usually consumed in the form of a warm drink, prepared at a temperature of 65–80 °C [2], similarly to tea, but usually brewed in boiling or slightly cooled water [50]. In addition, unlike tea, Yerba Mate is brewed many times (depending on consumer preferences) [16]. Which is why, in this study, the effect of the temperature, optimal for brewing Yerba Mate (70 °C) and optimal for brewing most teas (100 °C), and brewing numbers (1-time and 2-times) on the organoleptic, physicochemical and bioactive properties of Yerba Mate infusions obtained in this way were evaluated.

**Figure 2:**
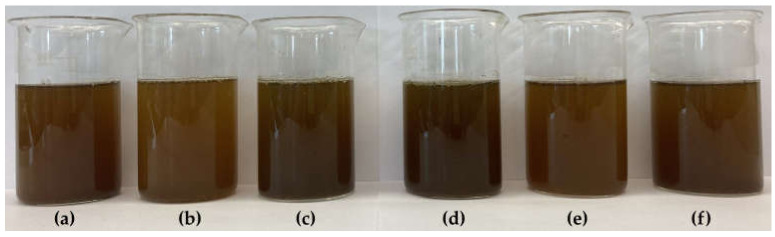
General appearance of filtered Yerba Mate infusions obtained as a result of single brewing at 70 °C and 100 °C: YM-A-1-70 (**a**), YM-B-1-70 (**b**), YM-C-1-70 (**c**), YM-A-1-100 (**d**), YM-B-1-100 (**e**), YM-C-1-100 (**f**).

**Figure 3:**
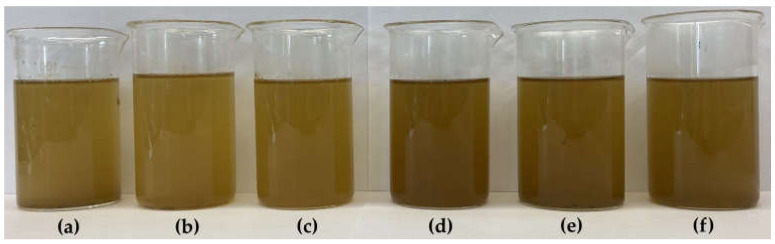
General appearance of filtered Yerba Mate infusions obtained as a result of double brewing at 70 °C and 100 °C: YM-A-2-70 (**a**), YM-B-2-70 (**b**), YM-C-2-70 (**c**), YM-A-2-100 (**d**), YM-B-2-100 (**e**), YM-C-2-100 (**f**).

Figure 2 shows the general appearance of filtered Yerba Mate infusions prepared at 70 °C and 100 °C after the first brewing, while Figure 3 shows the infusions obtained after the second brewing at both temperatures.

In the organoleptic evaluation, the infusions obtained as a result of the first brewing (Figure 2) were characterized by an intense green-brown color, which they clearly lost during the double brewing (Figure 3).

Yerba Mate infusions obtained after single brewing were characterized by an intense, smoky aroma and noticeable woody notes. The taste of YM-A and YM-B infusions was from the beginning expressive, intense, sour, slightly tart, with a hint of bitterness reminiscent of the aftertaste of fresh walnut peel, while the intensity of the YM-A flavor faded quickly, clearly softening in the second brewing. YM-B infusion retained its flavor much longer, losing little on the second brewing, which made it a perfect Mate for multiple brews. On the other hand, the YM-C infusion was initially quite smooth in taste, quickly becoming creamy or even thick, with vegetable, sweet and sour and smoky notes.

After preparing the infusions, it was noticed that the samples differed in the intensity of the bitter, sour and smoked taste, with the infusions prepared at a higher temperature (100°) being more bitter than those brewed at 70 °C. YM-A-1-100 infusion was characterized by the most intense bitter taste.

All the infusions obtained during the second brewing, both at 70 °C and 100 °C, became clearly softened, less intense, more delicate and watery in taste. The infusion that retained its taste and aroma from the first brewing to the greatest extent was YM-C.

In the organoleptic evaluation, the infusions obtained as a result of double brewing (Figure 3) were characterized by a clearly weaker color intensity and its much less color differentiation, as well as lighter shades or greater clarity than infusions obtained during single brewing (Figure 2). Therefore, in Yerba Mate infusions prepared at different temperatures (70 °C and 100 °C), color parameters in the L*a*b* color space, various color functions, i.e., browning index (IB) and total color difference (Δ*E*) were evaluated, and the obtained results are presented in Table 2 (single brewing) and in Table 3 (double brewing).

All infusions obtained as a result of double brewing (Table 3) were characterized by much greater brightness (by almost 60%) than those brewed once (Table 2). The L* parameter, was on average 36.46 ± 4.46 and 57.55 ± 1.65 (for double and single infusions, respectively) with samples brewed once at a lower (70 °C) temperature being slightly lighter (on average 38.13 ± 5.06) than those brewed in higher (100 °C) temperature (average 34.78 ± 3.22). In addition, the L* parameter in the case of infusions brewed twice showed much less variability than in infusions brewed once.

Considering the single brewing (Table 2), the darkest shade was found in the YM-A-1-100 infusion (30.60 ± 0.20), while the YM-B-1-70 infusion was significantly (*p* ≤ 0.05) the brightest (43.80 ± 1.70). The lowest L* value in infusions brewed twice (Table 3) were recorded in YM-C-2-70 (55.05 ± 0.25), higher (*p* ≤ 0.05) in YM-A-2-100, YM-B-2-70, YM-B-2-100 and YM-C-2-100, while the brightest was YM-A-2-70 infusion (60.00 ± 0.20).

Comparing the number of Yerba Mate brews effect on the color parameters, the greatest differences were found in the case of chromatic coordinate a*. Yerba Mate brewed once (Table 2) showed positive (+a*) values (on average 13.57 ± 2.31), and therefore a predominance of red shades, while those brewed twice (Table 3) showed negative (−a*) values, which means that they were dominated by shades of green (average −1.65 ± 0.89).

Yerba Mate infusions with the highest (*p* ≤ 0.05) red color saturation were YM-A-1-100 and YM-C-1-70 (average 15.56 ± 0.28), followed by YM-B-1-100 and YM-C-1-100 (mean 14.43 ± 0.62) and YM-A-1-70 (12.10 ± 0.40). The lowest (*p* ≤ 0.05) degree of saturation with red color was characteristic of the YM-B-1-70 infusion (9.35 ± 0.15), which was at the same time the lightest among those brewed once.

In the case of double brewing (Table 3), all Yerba Mate infusions obtained negative a* values, which means that they were characterized by a predominance of green (−a*) over red (+a*) shades, and the highest share of green color was noted in YM-C-2-100 (−2.85 ± 0.05), lower in YM-B-2-70 and YM-A-2-70 infusions. The smallest color shift towards green shades was found in YM-B-2-100 and YM-A-2-100 infusions (average −0.64 ± 0.09).

Taking into account the chromatic coordinate b*, the instrumental color measurement showed the smallest differences between Yerba Mate brewed once and twice. This parameter for all infusions had positive values (+b*), meaning that they had more yellow (+b*) than blue (−b*) shades, with a slightly larger color shift towards yellow in a double infusion (average 44.25 ± 4.00) than in a single one (average 41.34 ± 3.90).

In the case of single-brewed infusions, higher saturation with yellow shades was noted in samples brewed at a lower temperature (70 °C), for which b* value was 43.27 ± 2.52, with the highest (*p* ≤ 0.05) values in YM-C-1-70 and YM-B-1-70 (mean 44.93 ± 0.48). Yerba Mate brewed once at 100 °C reached an average of 39.42 ± 4.20 for this parameter, with the lowest (*p* ≤ 0.05) value for YM-A-1-100 (34.05 ± 0.25). In the case of double brewing, the opposite trend was found. When brewing at a higher temperature (100 °C), higher b* values were obtained (average 45.88 ± 4.54) than at a lower temperature (70 °C) (average 42.62 ± 2.72). The highest (*p* ≤ 0.05) b* values were recorded in YM-A-2-100 and YM-B-2-100 (average 48.90 ± 0.36), and the lowest in YM-B-2-70 (39.15 ± 0.35). The available literature does not contain data on a detailed analysis of color parameters in Yerba Mate infusions prepared in different thermal conditions, i.e., brewed at 70 °C and 100 °C, or obtained during a single and double brewing process.

The research showed significant (*p* ≤ 0.05) differences in the total color difference (Δ*E*), with definitely greater color changes compared to the dried Yerba Mate samples during a single (average 28.46 ± 9.60) than a double (average 20.43 ± 5.04) of the brewing process. Single-brewed Yerba Mate infusions (Table 2) were more than 3 times darker than the dried samples, because the average *BI* was 300.93 ± 38.77 and 93.16 ± 29.59, respectively (Table 1). The highest (*p* ≤ 0.05) Δ*E* values were found in YM-C-1-70 infusion (41.66 ± 0.37), which was also characterized by a very high *IB* (316.68 ± 2.24). Double-brewed Yerba Mate infusions (Table 3) showed an average of almost 30% lower Δ*E* values than those brewed once and were almost 2.5 times brighter (the average *IB* was 125.26 ± 22.62). In the case of double-brewed Yerba Mate infusions, the largest changes (*p* ≤ 0.05) in color compared to dried samples occurred in the YM-B-2-100 infusion, with the highest Δ*E* (28.81 ± 0.70) (Table 3).

In the infusions obtained as a result of different temperature conditions (brewing at 70 °C and 100 °C) and the number of infusions (1-fold and 2-fold), basic physicochemical parameters such as pH, total soluble solids (°Brix) and osmolality were assessed and the results are shown in Table 4 (single brewing) and Table 5 (double brewing).

Yerba Mate infusions were characterized by a similar pH, with a single brewing the pH was on average 5.44 ± 0.07, and with a double brewing it was slightly higher (5.56 ± 0.08 on average). The highest (*p* ≤ 0.05) pH was found in the same Yerba Mate infusion (YM-A), during single (5.56 ± 0.03) (Table 4) and double brewing at 70 °C (5.72 ± 0.01) (Table 5). These pH results are confirmed by other authors. Alcarraz et al. (2021) [57] obtained the pH of Yerba Mate infusions from 4.5 to 5.0, while others at the level of about 4.3 ± 0.01 [58].

The tested Yerba Mate infusions were characterized by an acidic reaction (pH < 7.0), which could indicate good microbiological quality of the dried products and their greater durability due to the inhibition of the development of microorganisms responsible for product rotting during improper storage conditions [56].

The average soluble solids content in single-brewed Yerba Mate infusions (Table 4) was 2.08 ± 0.06%, in double-brewed (Table 5) it was almost 3 times lower (0.72 ± 0.09%). The highest °Brix was found in YM-A-1-100 (2.17 ± 0.06%), and significantly lowest (*p* ≤ 0.05) in YM-B-2-70 (0.60 ± 0.00%). The obtained °Brix values were consistent with the results of other authors who, in studies on the phytochemical composition of Yerba Mate (*chimarrão*) extracts, showed °Brix at the level of 1.29 ± 0.19% to 3.47 ± 0.38% [59].

The tested infusions were also characterized by very low osmolality, with over 3.5 times higher in single brewing (Table 4) (mean 49.44 ± 3.60 mOsm/kg•H_2_O) compared to double brewing of Yerba Mate (Table 5) (mean 14.00 ± 2.33 mOsm/kg•H_2_O). The highest (*p* ≤ 0.05) osmolality, similarly to °Brix, was found in a single-brewed YM-A-1-100 infusion (55.33 ± 0.58 mOsm/kg•H_2_O), whereas the lowest (*p* ≤ 0.05) was observed in both, single (45.33 ± 0.58 mOsm/kg•H_2_O) and double Yerba Mate brewing (11.67 ± 0.58 mOsm/kg•H_2_O) at 70 °C. The osmolality of the tested Yerba Mate infusions obtained by double brewing was similar to the results of other authors, who showed the value of this parameter at the level of 12.8 mOsm/kg•H_2_O in Yerba Mate infusions [57].

The available literature lacks of data on the effect of brewing conditions, i.e., temperature and number of brewing, on physicochemical parameters such as pH, °Brix or osmolality in Yerba Mate infusions. Considering the very low °Brix and osmolality values, the tested infusions could be classified as hypotonic drinks, next to products such as mineral or spring waters [60]. Their absorption from the digestive tract into the cells takes place very quickly, leading to equally rapid hydration of the body [61].

### 2.2. Bioactive Properties of Different Yerba Mate Infusions

This study investigated the influence of brewing conditions on the content of bioactive ingredients and the antioxidant activity of Yerba Mate infusions.

#### 2.2.1. Tannins

**Figure 4:**
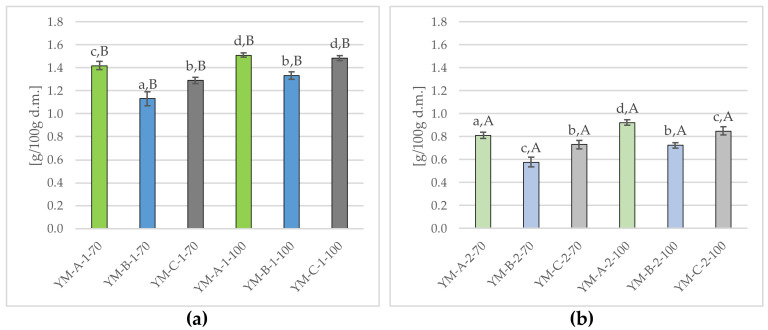
Tannin content (g/100 g d.m.) in Yerba Mate infusions obtained as a result of single (**a**) and double (**b**) brewing at 70 °C and 100 °C. Mean values marked in bars by different letters differ significantly (Duncan’s test*, p* ≤ 0.05). (^a–d^)—values for different samples and brewing temperature in single (**a**) and double (**b**) brewing; (^A–B^)—differences between single (**a**) and double (**b**) brewing for each Yerba Mate sample.

Figure 4 shows the tannin content in Yerba Mate infusions obtained as a result of single (a) and double (b) brewing at 70 °C and 100 °C.

The tannin content in single-brewed Yerba Mate infusions ranged from 1.13 ± 0.06 up to 1.51 ± 0.02 g/100 g d.m. (mean 1.36 ± 0.14 g/100 g d.m.) (Figure 4a), with the tested samples significantly (*p* ≤ 0.05) differing in this parameter. The lowest tannin concentration was found in the YM-B-1-70 infusion, significantly higher in YM-C-1-70 and YM-B-1-100 infusions (mean 1.31 ± 0.04 g/100 g d.m.), while the highest one was in YM-C-1-100 and YM-A-1-100 (mean 1.50 ± 0.02 g/100 g d.m.). In double-brewed infusions, the tannin content decreased by about 43.4%), and the average content of these components was 1.36 ± 0.12 g/100 g d.m. As in the case of single-brewed samples, the lowest (*p* ≤ 0.05) tannin content was found in YM-B-2-70 (0.58 ± 0.04 g/100 g d.m.) and the highest in YM-A-2-100 (0.92 ± 0.03 g/100 g d.m.). In the available literature, there are no reports on the influence of conditions such as temperature, time and the number of infusions on the content of tannins in Yerba Mate infusions. Studies on the tannin content in Yerba Mate in general are also isolated [19,62].

In the studies by Pizarro et al. (1994) [62] on the tannin content in herbal teas commonly consumed in Chile and other Latin American countries, the amount of these ingredients was higher (11.7 g/100 g d.m.) than in our study. In turn, in the study by Frizon and Nisgoski (2020) [19], the content of these ingredients was similar to that obtained in this study and ranged from 0.38 up to 3.20 g/100 g d.m. (depending on growing and storage conditions of Yerba Mate). However, in both cited studies, other techniques were used to determine the tannin content (i.e., Folin-Denis in the study by Pizarro et al. (1994) [62] or the Prince method in the study by Frizon and Nisgoski (2020) [19], not the titration method (as in experiment), which makes it very difficult to compare quantitative results.

The conducted research also showed the effect of temperature on the tannin content, both in single (Figure 4a) and double (Figure 4b) Yerba Mate infusions. A higher brewing temperature (100 °C) was accompanied by a higher tannin concentration compared to infusions obtained in 70 °C, i.e., by approx. 12.5% and 16.9% (single and double brewing). However, the available literature does not contain any data on the effect of temperature on the content of these bioactive ingredients in Yerba Mate infusions. Nevertheless, there are (albeit single) reports on the effect of brewing conditions on the tannin content in tea [18], an equally common beverage consumed in the form of hot infusions [50].

Dmowski et al. (2011) [18] showed significant differences in the content of tannins in various tea infusions depending on the temperature of the water used for their preparation. The tannin content in teas brewed at a higher (90 °C) temperature ranged from 3.33 to 9.03 g/100 g and was significantly higher than for infusions prepared at a lower (70 °C) temperature (from 1.63 up to 1.97 g/100 g d.m.) [18]. Despite the definitely higher tannin content in tea compared to Yerba Mate infusions, the tendency of changes in the amount of these components under the influence of temperature in various types of tea was analogous to our study. Dmowski et al. (2011) [18] also showed significant differences in the tannin content depending on the region of tea origin, with teas from Vietnam and Mozambique having the highest tannin content, from Argentina lower, and from Malawi the lowest [18]. In other study, Jyotismita et al. (2015) [63] showed the differences in tannin content depending on tea production technology, especially on the degree of leaves fermentation (oxidation) [63]. The highest tannin content was found in black tea (mean 13.36% in dry matter), lower in Oolong, and the lowest in green tea (mean 2.65% in dry matter). Black teas are the result of complete fermentation of tea leaves, leading to the formation of a typical brown-black color, resulting from the enzymatic oxidation of catechins present in tea leaves. Oolong teas are made as a result of incomplete fermentation, while green teas are produced without this process. Therefore, an important step in the production of green teas is the phenol oxidase deactivation by using high-temperature heat treatment (steaming or roasting), which allows the green tea color to be preserved [63].

Comparing the quoted results of the tannin content in various tea types with the results obtained in this work, it can be concluded that Yerba Mate samples were the most similar to green teas. Also, in the Yerba Mate production technology, similarities can be seen. Similarly to the green tea production process, the first (after harvesting holly leaves) is the heat treatment process (bleaching). It consists in short (2–3 min) smoking at 500–600 °C in order to inactivate the enzymes responsible for oxidation and preserve the characteristic sensory features, i.e., the color, aroma and taste of the leaves [2,40,64]. As Frizon and Nisgoski (2020) [19] reported, the tannins content in Yerba Mate was significantly different at different stages of Paraguayan holly treatment. Fresh (green), unheated leaves had the lowest tannins content (from 0.39 to 0.56 g/100 g d.m.), significantly higher-leaves subjected to bleaching (from 0.96 up to 2.35 g/100 g d.m.) and the highest (from 1.59 to 3.20 g/100 g d.m.) leaves subjected to preliminary drying at high temperatures.

Also grinding had an impact on the content of these ingredients, changing the tannin concentration (from 2.21 to 2.25 g/100 g d.m.) as well as storing (from 1.96 to 2.25 g/100 g d.m.) of ready-made Yerba Mate blends [19]. In addition, the same authors showed that the tannin content varied depending on the growing conditions of Paraguayan holly, with products obtained from conventional cultivation (usually open areas with more exposure to light) having higher tannin content than those from organic cultivation (regions with more shade) at various stages of leaf processing and Yerba Mate production [19].

Tannins present in Yerba Mate, similarly to those in tea, are responsible for the characteristic bitterness and astringency of these infusions [18,19]. According to the literature, the optimum water temperature for brewing Yerba Mate is usually between 65 °C and 80 °C, and the higher the water temperature, the stronger and bitterer the brew obtained [2]. The organoleptic evaluation of the infusions carried out in this study showed that YM-A-1-100 infusion was marked by the greatest bitterness and astringency, which at the same time had a significantly (*p* ≤ 0.05) highest tannin content (Figure 4a). It can be concluded that tannins present in Yerba Mate and the conditions for preparing the infusions play an important role in shaping the organoleptic characteristics and bioactive properties of the beverages obtained in this way.

#### 2.2.2. Caffeine

**Figure 5:**
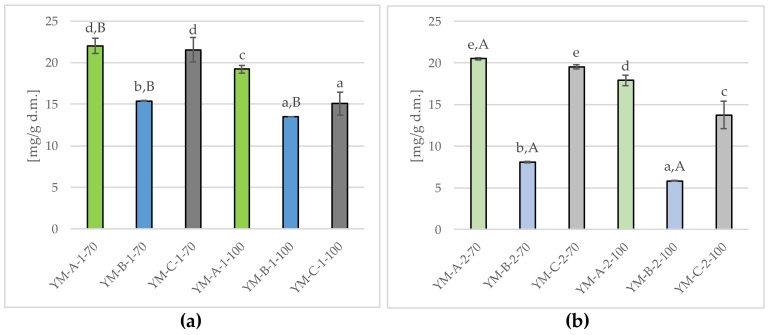
Caffeine content (mg/g d.m.) in Yerba Mate infusions obtained as a result of single (**a**) and double (**b**) brewing at 70 °C and 100 °C. Mean values marked in bars by different letters differ significantly (Duncan’s test*, p* ≤ 0.05). (^a–e^)—values for different samples and brewing temperature in single (**a**) and double (**b**) brewing; (^A–B^)—differences between single (**a**) and double (**b**) brewing for each Yerba Mate sample.

Figure 5 shows the caffeine content in Yerba Mate infusions obtained as a result of single (a) and double (b) brewing at temperatures of 70 °C and 100 °C.

The caffeine content in the infusions obtained as a result of single brewing ranged from 13.51 ± 0.02 mg/g d.m. up to 22.02 ± 0.95 mg/g d.m. (average 17.79 ± 3.49 mg/g d.m.) (Figure 5a), and the tested samples differed significantly (*p* ≤ 0.05) in terms of this component. The lowest caffeine content was found in YM-B-1-100, YM-C-1-100 and YM-B-1-70 infusions (average 14.66 ± 1.12 mg/g d.m.), significantly higher in YM-A-1-100 (19.20 ± 0.48 mg/g d.m.), and the highest in YM-C-1-70 and YM-A-1-70 (average 21.79 ± 1.16 mg/g d.m.). As a result of double brewing, a reduction in caffeine content was found. The average content of this alkaloid in twice-brewed samples was 14.28 ± 5.80 mg/g d.m. and (similarly to those brewed once) the lowest (*p* ≤ 0.05) content was found in YM-B-2-100 infusion (5.86 ± 0.01 mg/g d.w.), while the highest in YM-A-2-70 (20.50 ± 0.16 mg/g d.w.) (Figure 5b).

According to the literature, the caffeine content in Yerba Mate is usually in the range of 1 to 2% in dry matter [2,53] and can range from approx. 25 to 175 mg/g d.m. [6,26,65], which was confirmed by the research conducted in this study (0.6% to 2.2% in dry matter).

In the literature, there are single studies on the influence of Yerba Mate brewing conditions, such as temperature, time and number of brewings, on the caffeine content in Yerba Mate [53,66]. Meinhart et al. (2010) [53] showed a significant decrease in caffeine concentration during multiple brewing of Yerba Mate at 75 °C for 30 s, i.e., from 13.7–26.4 mg/100 mL of infusion (during a single brewing) to 1.5–6.6 mg/100 mL in 30-brewed Yerba Mate *chimarrão*. These authors showed a similar tendency for multiply brewed Yerba Mate *tereré* at a lower (11 °C) temperature at the same time (30 s). In this case, the caffeine content in single infusions was approx. 35.8 mg/100 mL, while after 30 infusions it was only 2.9 mg/100 mL [53]. This tendency was also confirmed by the research conducted in this study, as double brewing resulted in a reduction in the caffeine content by approx. 19.7% compared to single-brewed Yerba Mate infusions.

Kruszewski et al. (2012) [66] also showed that the caffeine content in Yerba Mate infusions increases with the extension of the brewing time, from 15 mg/100 mL of the infusion prepared in 15 min to 25.8 mg/100 mL in 60 min at 70 °C, while for Yerba Mate brewed in boiling water (100 °C) from 11.8 mg/100 mL (15 min.) to 15.8 mg/100 mL (60 min.). The research of these authors also shows that the concentration of caffeine in infusions prepared at a higher temperature (100 °C) is significantly lower compared to infusions obtained at 70 °C, which was confirmed by our research. Yerba Mate samples brewed once at 100 °C had lower caffeine content, on average by 1.87 ± 0.04 mg/g d.m. (YM-B-1), 2.82 ± 0.75 mg/g d.m. (YM-A-1) and 6.48 ± 0.71 mg/g d.m. (YM-C-1) than those brewed at 70 °C. A similar relationship was found in double-brewed Yerba Mate infusions, for which the caffeine concentration was lower, on average by 2.23 ± 0.04 mg/g d.m. (YM-B-2), 2.60 ± 0.55 mg/g d.m. (YM-A-2) and 5.78 ± 1.52 mg/g d.m. (YM-C-2) in the case of samples steamed at a higher temperature, i.e., in boiling water (100 °C) than at 70 °C.

The results of the qualitative and quantitative analysis of selected bioactive compounds, i.e., carotenoids, phenolic acids and flavonoids identified by HPLC in Yerba Mate infusions are presented in Table 6 (single brewing at 70 °C and 100 °C) and in Table 7 (double brewing at 70 °C and 100 °C).

#### 2.2.3. Carotenoids

The tested Yerba Mate samples were characterized by a high content of carotenoids, with the sum of identified ingredients on average 12.90 ± 0.44 μg/g d.m. in single-brewed (Table 6) and 12.67 ± 0.62 μg/g d.m. in double-brewed (Table 7) infusions. The highest (*p* ≤ 0.05) content of these compounds was found in YM-A samples brewed at 100 °C, both during single (YM-A-1-100) and double (YM-A-2-100) brewing (13.58 ± 0.08 μg/g d.w. and 13.44 ± 0.17 μg/g d.w., respectively), the lowest (*p* ≤ 0.05) in YM-B samples brewed at 70 °C, both during a single brewing (YM-B-1-70), as well as double brewing (YM-B-2-70) (12.20 ± 0.08 μg/g d.m. and 11.84 ± 0.08 μg/g d.m., respectively).

HPLC analysis identified three carotenoid compounds, i.e., lutein, α-carotene and β-carotene. The dominant ingredient was lutein, 63.79 ± 1.35% (single brewing) and 69.36 ± 1.27% (double brewing) of total carotenoids. α-carotene had a much smaller share in this fraction of bioactive ingredients (26.70 ± 0.65% and 25.56 ± 1.06%), while β-carotene accounted for only 9.50 ± 1.95% and 5.07 ± 0.69% (for samples brewed once and twice, respectively). According to the literature, lutein is the main carotenoid from the xanthophyll group, present in green leaves and green vegetables [67,68].

There are few studies in the available literature on the content and profile of carotenoids in Yerba Mate, and the existing ones indicate a very large diversity of these ingredients. For example, in studies on the influence of a controlled atmosphere on the quality of Yerba Mate during long-term (10 months) storage, Thewes et al. (2016) [69] showed the carotenoid content ranging from 92.93 μg/g f.w. up to 107.9 μg/g f.w. (at the beginning of storage tests), from 152.6 μg/g f.w. to 465.9 μg/g f.w. (in various shelf life periods, 1–4 weeks), or from 139.7 μg/g f.w. to 589.9 μg/g f.w. (after 10 months of storage), depending on the type of crop and different storage conditions of Yerba Mate. However, these tests were not carried out in infusions, but in Yerba Mate samples stored after harvest.

The use of different brewing conditions for Yerba Mate slightly influenced the content of identified total carotenoids (lower by less than 2% in double-brewed infusions). However, it is worth paying attention to the profile of these ingredients. Both in infusions brewed once (Table 6) and twice (Table 7), the content of total carotenoids was higher at higher temperature (100 °C). A similar tendency was shown by da Silveira et al. (2016) [68] for lutein in water extracts in four types of Yerba Mate (commercial products differing, among others, in the degree of granulation). The degree of lutein migration into aqueous extracts increased with increasing temperature. In traditional Yerba Mate, brewed at 67 °C, 75 °C, 85 °C and 95 °C, it increased from 1.99 ± 0.26 μg/100 mL to 2.13 ± 0.72 μg/100 mL. In turn, in the coarse-ground Yerba Mate, lutein content increased from 2.35 ± 0.57 μg/100 mL to 5.00 ± 1.95 μg/100 mL of extract [68]. In our research, such a relationship was observed for lutein during double brewing of Yerba Mate (Table 7).

#### 2.2.4. Phenolic Acids and Flavonoids

According to the conducted research, Yerba Mate infusions were characterized by a high content of phenolic compounds, with the sum of identified ingredients on average 75.44 ± 8.46 mg/g d.m. in single-brewed (Table 6) and 61.40 ± 9.22 mg/g d.m. in double-brewed (Table 7) Yerba Mate samples. The obtained results coincide with those of other authors, who showed the content of these compounds in Yerba Mate at the level of 80-97 mg/g d.m. [25,70]. According to the literature, the fraction of polyphenolic compounds in Yerba Mate leaves may constitute from 7 to 10% of dry matter [71].

Based on the results, it can be concluded that in both once (Table 6) and twice (Table 7) brewed infusions, the main fraction of phenolic compounds were phenolic acids, the share of which (in all identified ingredients) was 92.87 ± 1.76% and 94.04 ± 0.90%, respectively. In the infusions obtained as a result of single brewing, *p*-coumaric acids (45.1%) and chlorogenic acids (31.5%) dominated among the identified phenolic acids. *t*-cinnamic (9.5%) and caffeic (8.7%) acids had a much smaller, and gallic (2.0%), ferulic (2.0%) and salicylic (1.2%) acids had the smallest share. In the case of infusions obtained as a result of double brewing, the dominant acids were *p*-coumaric (31.9%) and caffeic (28.9%), while the lower share had chlorogenic (13.2%), *t*-cinnamic (13.1%), and ferulic (7.7%) acids. The smallest ones were salicylic (3.0%) and gallic (2.1%) acids.

The obtained results were partially confirmed by other authors. Bravo et al. (2007) [25] found that phenolic acids constitute (as in our work) over 90% of the content of all phenolic compounds, with quinic acid dominating. According to the authors, 80% of all compounds were 9 main phenolic components belonging to hydroxycinnamoyl quinic acid esters and flavonol glycosides [25]. Research by Piovezan-Borges et al. (2016) [72] also confirmed that the main fraction of phenolic compounds are hydroxycinnamates, but mainly these are caffeic acid esters and other hydroxycinnamic acids, such as ferulic, *p*-coumaric acid, or caffeic acid, constituting up to 95% of the content of all phenolic compounds. The remaining 5% of the PC fraction consists of flavonols [72]. Piovezan-Borges et al. (2016) [72] also confirmed that the main fraction of phe-nolic compounds are hydroxycinnamates, but mainly these are caffeic acid esters and other hydroxycinnamic acids, such as ferulic, *p*-coumaric or caffeic acids, constituting up to 95% of all phenolic compounds. The remaining 5% of the phenolic compounds consists of flavonols fraction [72]. Also Cheminet et al. (2021) [73] showed over 90% share of phenolic acids in the identified phenolic compounds, showing the highest share of caffeoylshiquimic (70%), much lower for feruroylquinic acids (7.0%) and dicaffeoylquinic (5.5%) acids [73]. Similarly, in the study by Mateos et al. (2017) [70], the main group of phenolic compounds were phenolic acids with a predominant share in this fraction of caffeoylquinic acids, of which 3-caffeoylquinic, 5-caffeoylquinic and 4-caffeoylquinic acids were the dominant compounds (26.8–28.8%, 21.1–22.4% and 12.6–14.2% of all phenolic compounds, respectively) [70]. Also, Konieczyński et al. (2017) [50], among the main phenolic compounds, mentioned phenolic acids, including caffeic (from 6.92 ± 2.22 mg/g d.m. to 9.96 ± 0.22 mg/g d.m.), ferulic (from 1.12 ± 0.00 mg/g d.m. up to 1.48 ± 0.02 mg/g d.m.), gallic (from 0.45 ± 0.01 mg/g d.m. to 0.49 ± 0.09 mg/g d.m.) and *p*-coumaric (from 0.06 ± 0.01 mg/g d.m. to 0.09 ± 0.00 mg/g d.m.) acids [50].

HPLC analysis showed that the second significant group of phenolic compounds in the tested Yerba Mate samples were flavonoids. The average concentration of these compounds in single-brewed (Table 6) and double-brewed (Table 7) infusions was 5.47 ± 1.78 mg/g d.m. and 3.64 ± 0.76 mg/g d.m., which represented 7.13 ± 1.76% and 5.96 ± 0.90% of all phenolic compounds identified by this method, respectively. Of the four identified flavonoids in single-brewed Yerba Mate infusions, two dominated, i.e., glycoside-3-*O*-quercetin (48.3%) and rutoside-3-*O*-quercetin (29.0%), while myricetin (18.4%) and apigenin (4.3%) had a smaller share (Table 6). In turn, in double-brewed Yerba Mate infusions, the dominant flavonoid was rutinoside-3-*O*-quercetin (45.5%), a much smaller share had glycoside-3-*O*-quercetin (29.4%), then myricetin (22.5%) and apigenin (2.6%) (Table 7).

The obtained results were confirmed in studies by other authors. According to the literature, in terms of concentration, flavonoids constitute the second fraction of phenolic components in Yerba Mate (up to 10% of all phenolic compounds) [25,26,27,46,70,73].

The most frequently identified flavonoids in Yerba Mate include: rutinoside-3-*O*-quercetin, glycoside-3-*O*-quercetin, rhamnoside-3-*O*-quercetin, 3-rhamnoside and 3-glycoside of kaempferol, luteolin diglycoside, rutin, catechin, quercetin, myricetin and apigenin [2,25,26,27,46,50,70], as well as rutoside and astragalin [23], but the literature does not agree on the compounds dominant in this fraction. Some studies show the dominant share (up to 90% of identified flavonoids) of rutin [25,70], others of rutoside [23], still others of quercetin [50], or rutinoside-3-*O*-quercetin [73].

The conducted research showed significant (*p* ≤ 0.05) differences in the profile and content of individual fractions of polyphenolic compounds between single-brewed Yerba Mate samples (Table 6). The highest (*p* ≤ 0.05) content of phenolic acids and flavonoids was found in the YM-A-1- 70 (78.95 ± 0.49 mg/g d.m. and 7.71 ± 0.09 mg/g d.m., resp.), lower in YM-C-1-70 (75.29 0.92 mg/g d.m. and 7.16 ± 0.06 mg/g d.m., resp.), the lowest (*p* ≤ 0.05) in YM-B-1-100 (57.10 ± 0.61 mg/g d.m. and 3.54 ± 0.03 mg/g d.m., resp.). A similar trend occurred in double-brewed Yerba mate samples (Table 7). The highest total phenolic acids and flavonoids was found in YM-A-2-70 (65.91 ± 0.62 mg/g d.m. and 5.18 ± 0.03 mg/g d.m., resp.) and YM-C-2-70 (65.23 ± 0.18 mg/g d.m. and 3.89 ± 0.00 mg/g d.m., resp.), significantly lower in YM-A-2-100 (59.31 ± 0.01 mg/g d.m. and 3.38 ± 0.03 mg/g d.m., resp.), and the lowest (*p* ≤ 0.05) in YM-B -2-100 (40.32 ± 0.21 mg/g d.m. and 3.06 ± 0.03 mg/g d.m., resp.).

The available literature does not contain any data on the impact of different brewing conditions, i.e., the number of brewing or temperature, on the profile and content of phenolic acids and flavonoids in Yerba Mate. It is worth noting here, that YM-A and YM-C samples brewed at a lower temperature (70 °C) were characterized by a higher content of phenolic acids, i.e., chlorogenic, caffeic, ferulic, salicylic and *t*-cinnamic acids. In the case of gallic, chlorogenic and *p*-coumaric acid, this tendency was also shown by YM-B sample. In turn, in the case of flavonoids, all Yerba Mate samples brewed at a lower temperature (70 °C) showed a higher content of each of these compounds compared to samples brewed in boiling water (100 °C). The exception was the YM-A sample, where no such relationship was found only in the case of myricetin. Interestingly, the concentration of this ingredient was the highest (*p* ≤ 0.05) in YM-A-1-100 sample (brewed at 100 °C) (1.64 ± 0.02 mg/g d.m.) among all the analyzed samples (Table 6). In the case of infusions obtained as a result of double brewing (Table 7), these relationships were not so clear and were characterized by greater diversity in the case of individual bioactive compounds.

Taking into account the huge diversity of phenolic compounds, complex biosynthetic pathways and metabolism of individual components, as well as the significant impact of both genetic, environmental and processing factors on the profile of phenolic compounds in Yerba Mate [50,70,73], further research is necessary, in particular to precisely determine the qualitative and quantitative composition of individual phenolic compounds, for a better understanding of their bioactivity in the case of consumption of Yerba Mate as a potential functional food [70,73].

#### 2.2.5. Total Polyphenols

**Figure 6:**
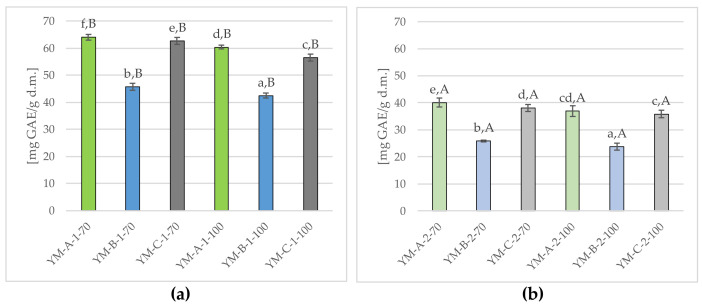
Total polyphenol content (mg GAE/g d.m.) in Yerba Mate infusions obtained as a result of single (**a**) and double (**b**) brewing at 70 °C and 100 °C. Mean values marked in bars by different letters differ significantly (Duncan’s test*, p* ≤ 0.05). (^a–f^)—values for different samples and brewing temperature in single (**a**) and double (**b**) brewing; (^A–B^)—differences between single (**a**) and double (**b**) brewing for each Yerba Mate sample.

Figure 6 shows the total polyphenol content in Yerba Mate infusions obtained as a result of single (a) and double (b) brewing at 70 °C and 100 °C.

Studies have shown a high content of total polyphenolic compounds, both in infusions obtained as a result of single brewing (average 55.26 ± 8.51 mg GAE/g d.M.) (Figure 6a) and as a result of double brewing (average 32.19 ± 6.30 mg GAE/g d.m.) (Figure 6b), while the average content of these ingredients was significantly (*p* ≤ 0.05) lower (by approx. 40%) in the infusions obtained in the second brewing. Moreover, differences in the content of total polyphenols were found depending on the applied temperature. The total polyphenol content in the infusions obtained as a result of the first brewing (Figure 6a) at 70 °C was on average 57.47 ± 8.66 mg GAE/g d.m. and was slightly higher than in infusions obtained at 100 °C (53.04 ± 7.98 mg GAE/g d.m.). A similar trend was also observed after the second brewing (Figure 6b). Yerba Mate samples brewed at 70 °C were characterized by a slightly higher content of total polyphenols (34.69 ± 6.59 mg GAE/g d.m.) than those brewed at 100 °C (32.19 ±6.30 mg GAE/g d.m.).

According to the literature, the optimum temperature of water for brewing Yerba Mate, ensuring moderate bitterness, taste and aroma, is usually in the range of 65 °C to 80 °C [2]. However, in the available literature, there is little research on the effect of brewing temperature on the content of bioactive ingredients in Yerba Mate. In studies on the effect of water temperature of *chimarrão* infusions on the content of caffeic acid, five isomers of chlorogenic acid and rutin in two types of Yerba Mate (traditional and coarse), a significant effect of temperature on the content of these phenolic compounds was shown, with the highest concentration of all tested bioactive ingredients at 95 °C, approx. 30% lower at 65 °C, approx. 33% lower at 85 °C, and the lowest at 75 °C [22]. The authors explained this relationship with probably better solubility of these components in water at high (close to boiling) temperature [74]. In our study, an inverse relationship was found, but they concerned the total content of phenolic compounds and not changes in individual bioactive compounds.

The conducted studies also showed significant (*p* ≤ 0.05) differences in the total polyphenol content between all Yerba Mate samples brewed once (Figure 6a), with the highest content of these ingredients found in YM-A-1-70 (64.06 ± 1.11 mg GAE/g d.m.), lower in YM-C-1-70, YM-A-1-100, YM-C-1-100 and YM-B-1-70 and significantly (*p* ≤ 0.05) lowest in YM-B-1-100 (42.38 ± 0.94 mg GAE/g d.m.). Da Silveira et al. (2021) [22] showed a higher content of selected bioactive ingredients in coarse-grained Yerba Mate, compared to traditional Yerba Mate, which in turn was confirmed in this study, because the sample with the highest degree of fragmentation (YM-A) (Figure 1a) was characterized by the highest content of total polyphenols, in contrast to the YM-B sample, with the lowest degree of fragmentation and the highest share in the mixture of finely cut twigs and sticks (Figure 1b), showing the lowest content of these phytonutrients.

The results of total polyphenol content in single-brewed Yerba Mate infusions were confirmed by other authors [17,49,59]. Comparing various leaf extracts, Pinto et al. (2020) [59] showed from 55.23 ± 1.41 to 58.98 ± 0.10 GAE/g d.m. total polyphenols in aqueous extracts, from 54.48 ± 1.19 to 58.71 ± 0.82 GAE/g d.m. in ethanol extracts, while in ethanol:water (1:1) extracts, ranged from 58.88 ± 1.05 to 65.78 ± 1.54 mg GAE/g. Lower values (29.4 ± 0.4 to 39.7 ± 0.4 mg GAE/g d.m.) were obtained by Dmowski and Post (2018) [49] in single brewing Yerba Mate infusions. Similar, although slightly higher, values (71.6 ± 10.9 to 75.4 ± 5.7 mg GAE/g d.m.) were reported by Duarte et al. (2022) [17] in dried leaves of various Yerba Mate morphotypes. In other studies, total polyphenol content ranged from 76.47 to 84.88 GAE/g d.m. (average 80 mg GAE/g d.m.) [70], or from 81 to 97 mg GAE/g d.m. [25].

According to the literature, the content of total polyphenols varies depending on different parts of Yerba Mate, and compared to extracts from whole plants or twigs, the highest content is found in leaf extracts [6,25,26,27,44,46]. The total polyphenols content and other bioactive ingredients in Yerba Mate is also influenced by the methods of leaf treatment, e.g., the polyphenols in fresh leaves was 4.15 mg GAE/g d.m., in blanched “*zapecada*” 98.86 mg GAE/g d.m., in pre-dried 90.23 mg GAE/g d.m., dried-candchada 96.07 mg GAE/g d.m. and in forced aged leaves even 101.00 mg GAE/g d.m. [20]. Other authors showed a much higher share of total polyphenols in extracts from lyophilized commercial Yerba Mate products, reaching 143.2 ± 7.8 mg GAE/g of lyophilisate [75], while De Mejía et al. (2010) [26] in commercial samples of traditional Yerba Mate Instant produced from holly leaves from Argentina and Paraguay recorded the share of these phytochemicals even at the level of 244.9 ± 59.3 up to 321.8 ±74.1 mg GAE/g d.m.

Yerba Mate samples brewed twice (Figure 6b) were characterized by a slightly lower total polyphenol content, however, similarly to the first brewing, the highest (*p* ≤ 0.05) content of these components was found in the YM-A-2-70 (40.11 ± 1.72 mg GAE/g d.m.), and the lowest in YM-B-2-100 (23.80 ± 1.32 mg GAE/g d.m.). The double brewing process reduced the polyphenols content of around 22.78 ± 2.63 mg GAE/g d.m. (at 70 °C) and 20.85 ± 2.77 mg GAE/g d.m. (at 100 °C). It is worth noting, that despite the lowest content of polyphenols in the YM-B-2-100, it showed the smallest losses of these ingredients (on average by approx. 18.58 ± 1.81 mg GAE/g d.m.), in contrast to the YM-A-2-70 sample (with the highest content of polyphenols), in which the losses of these components after the second brewing were the highest and amounted to 23.97 ± 2.42 mg GAE/g d.m.

In the available literature, there are single studies on the influence of the multiplicity of Yerba Mate infusions on the content of polyphenolic compounds. Dmowski and Post (2018) [49] showed from 27.5 ± 02 up to 39.7 ± 0.4 mg GAE/g d.m. total polyphenols during the first brewing and a much higher (by about 55–59%) compared to the present study decrease in the content of these components during the second brewing (11.4 ± 0.4 to 17.1 ± 0.1 mg GAE/g d.m.). The next (third) brewing decreased polyphenols content (an average of another 45–67%), reaching only 3.7 ± 0.1 to 9.3 ± 0.2 mg GAE/g d.m. [49]. A similar trend was also shown by Colpo et al. (2016) [65] for commercial Yerba Mate products from Brazil, Argentina and Uruguay. Compared to the first brewing, the total polyphenol content in the second brewing decreased (similarly to our study) by about 40–45%, and each subsequent brewing led to further losses of these bioactive ingredients. In the Meinhart et al. (2010) [53] studies, the decrease in polyphenols content between the second and first brewing ranged from 48.6% to 55.5% in *chimarrão* infusions from various Yerba Mate commercial samples (smooth, traditional, native, course-ground), and each subsequent brewing reduced the content of these ingredients [53]. However, in the case of *tereré* (low-temperature brewing), the first 3–4 brewing resulted in polyphenols increase, and only the 4–5th brewing resulted in a significant decrease [53]. Based on the literature and conducted research, from the point of view of polyphenol content, apart from the number of infusions, the temperature and the method of infusion preparation are also important.

#### 2.2.6. Antioxidant Activity

**Figure 7:**
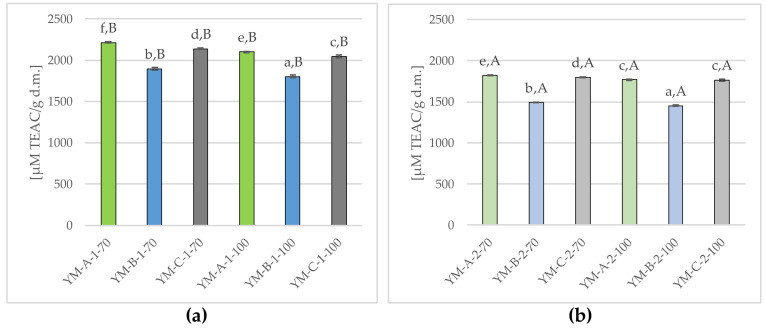
Antioxidant activity (μM TEAC/g d.m.) in Yerba Mate infusions obtained as a result of single (**a**) and double (**b**) brewing at 70 °C and 100 °C. Mean values marked in bars by different letters differ significantly (Duncan’s test*, p* ≤ 0.05). (^a–f^)—values for different samples and brewing temperature in single (**a**) and double (**b**) brewing; (^A–B^)—differences between single (**a**) and double (**b**) brewing for each Yerba Mate sample.

Considering that the tested Yerba Mate infusions provided a large amount of polyphenolic components, known as a strong antioxidants [9,16,17,20,23], the effect of single (a) and double (b) brewing at 70 °C and 100 °C on antioxidant activity in Yerba Mate was examined in this study and the results are presented in Figure 7. 

The infusions obtained during the first brewing (Figure 7a) were characterized by significantly higher antioxidant activity (mean 2031.98 ± 146.47 μM TEAC/g d.m.) compared to the infusions obtained after the second brewing (Figure 7b) (mean 1683.09 ± 155.34 μM TEAC/g d.m.), while (as in the case of total polyphenol content) slightly higher antioxidant potential was found in Yerba Mate samples brewed at 70 °C (mean 2081.69 ± 144.52 μM TEAC/g d.m. and 1704.24 ± 159.64 μM TEAC/g d.m., in the first and second brewing, respectively) than those brewed at 100 °C (on average 1982.26 ± 138.34 μM TEAC/g d.m. and 1661.93 ± 157.42 μM TEAC/g d.m., for the first and second brewing, respectively).

Among the single-brewed infusions (Figure 7a), the lowest (*p* ≤ 0.05) antioxidant activity was found in YM-B-1-100 (1801.44 ± 18.18 μM TEAC/g d.m.), higher values were noted for YM-B-1-70, YM-C-1-100, YM-A-1-100, YM-C-1-70, and the highest (*p* ≤ 0.05) in the YM-A-1-70 (2213.76 ± 7.10 μM TEAC/g d.m.). Double brewing of Yerba Mate caused a significant (*p* ≤ 0.05) reduction in the antioxidant properties. The lowest (*p* ≤ 0.05) antioxidant activity was recorded for YM-B-2-100 (1452.34 ± 9.09 μM TEAC/g d.m.) and the highest for YM-A-2-70 (1823.26 ± 5.95 μM TEAC/g d.w.) (Figure 7b).

According to the literature, Yerba Mate is characterized by a high antioxidant po-tential measured not only by the ability to deactivate ABTS^+•^ radicals [20,59,70,76], but also DPPH [16,17,23,45,46,48,59,64,75,77], or measured using FRAP [23,25,70,76] or ORAC [16,70,75,77]. Most studies used different research methods and analytical procedures (e.g., extraction, standard substances) and the results were expressed in different units, which makes it very difficult to compare them with the results obtained in this work.

Nevertheless, Mesquita et al. (2021) [16] showed similar antioxidant activity (from 1626 ± 0.22 to 1924 ± 0.16 μM TEAC/g) in Mate-Tea extracts, as did Mateos et al. (2018) [70] (2172.96 ± 169.15 μM TEAC/g to 2433.98 ± 241.71 μM TEAC/g) in various Yerba Mate commercial products. However, Mesquita et al. (2021) [16] also showed significantly higher antioxidant potential in *tereré* extracts (6194 ± 0.39 to 7726 ± 0.33 μM TEAC/g) or *chimarrão* (from 8246 ± 0.43 to 9106± 0.42 μM TEAC/g). In turn, in other studies, the antioxidant activity was much lower, i.e., from 830.6 ± 64.2 to 925.8 ± 99.5 μM TEAC/g d.m., depending on the different Yerba Mate morphotypes [17], from 410.50 ± 15.97 to 438.15 ± 18.39 μM TEAC/g d.m.) in various commercial Yerba Mate products, or it varied depending on the type of extraction used: from 297 ± 13 up to 353 ± 18 μM TEAC/g d.m. (aqueous extracts), from 275 ± 19 up to 352 ± 9 μM TEAC/g d.m. (ethanol extracts), or from 176 ± 7.0 up to 270 ± 11 μM TEAC/g d.m. (ethanol extracts: water, 1:1) [70].

This study showed that the antioxidant activity of Yerba Mate infusions significantly (*p* ≤ 0.05) decreased during the second brewing, both at 70 °C (approx. 18%) and 100 °C (approx. 16%) in compared to single infusions (Figure 7). A similar downward trend was found in the total polyphenol content, with the much higher losses, reaching almost 40%, both at 70 °C and 100 °C (Figure 6). Analogous trends have been confirmed in several studies by other authors [16,49,65].

Bravo et al. (2007) [25] in triple-brewed Yerba Mate samples showed very low antioxidant activity, i.e., from 1.56 ± 0.24 up to 1.71 ± 0.12 μM TEAC/g d.m. in infusions and from 1.48 ± 0.15 up to 1.81 ± 0.08 μM TEAC/g d.m. in extracts of various commercial Yerba Mate products. Dmowski and Post (2018) [49] showed that each of the three consecutive infusions significantly reduced the total polyphenol content and reduced (although not statistically significant) the antioxidant capacity of the infusions (measured using DPPH radicals). Also Colpo et al. (2016) [65] showed that the concentration of phenolic compounds significantly decreased with subsequent brewing, and the antioxidant activity (measured by the ability of the extracts to chelate Fe^2+^ iron, remove DPPH and NO radicals) decreased, but remained at a significant level. Considering that Yerba Mate is usually consumed in the form of multiple brewed infusions [16], the tendency to maintain significant antioxidant activity, even in fairly diluted infusions, is extremely important from the point of view of the pro-health potential, in particular the antioxidant activity, of this drink [65].

According to the literature, both the content of bioactive ingredients, including polyphenols, and the antioxidant potential of Yerba Mate, fall within very wide limits. The differences result from, among others: various brewing methods and conditions (including temperature, time or number of infusions) [16,45,48,49], botanical part of the plant, composition and degree of fragmentation of commercial available mixtures [6,25,26,27,44,46,47], from cultivar and morphotype [17,20,43], age of leaves, production technology and degree of Yerba Mate processing [3,20,21,42,45,46,48], also from origin [23] or plant growth conditions, soil type, climate and cultivation system [39,42].

**Figure 8:**
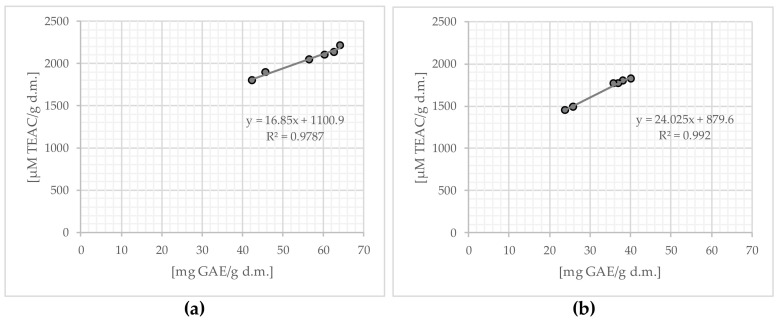
Relation between the content of total polyphenols (mg GAE/g d.m.) and antioxidant activity (μM TEAC/g d.m.) in Yerba Mate infusions obtained as a result of single (**a**) and double (**b**) brewing.

The conducted research showed a significant (*p* ≤ 0.05), positive relationship between the content of total polyphenols and antioxidant activity, both in infusions obtained as a result of a single (R^2^ = 0.9787 a) and double (R^2^ = 0.992) of brewing the tested samples of Yerba Mate, which is presented in Figure 8.

Bassani et al. (2013) [48] showed a relationship between the content of flavonoids and the antioxidant activity measured with the use of DPPH radicals (R^2^ = 0.9046 to R^2^ = 0.9972). Deladino et al. (2013) [75] found a correlation between chlorogenic acid vs. DPPH (R^2^ = 0.8429) and gallic acid vs. DPPH (R^2^ = 0.9807). Other authors have also confirmed that Yerba Mate has strong antioxidant properties, resulting, among others, from the high content of phenolic components [4,8,9,15,16,17,20,21,22,23,48,77].

The existence of a such relationship was confirmed by other authors. Mateos et al. (2018) [70] for infusions of various commercial Yerba Mate products, showed a significant, positive correlation between the content of total polyphenols and antioxidant activity measured both with the use of ABTS (R^2^ = 0.95), FRAP (R^2^ = 0.93)) and ORAC (R^2^ = 0.74). Blum-Silva et al. (2015) [45] found a relationship between total polyphenols and DPPH in water extracts from dried Paraguayan holly leaves, with the coefficient R^2^ = 0.9246, while Deladino et al. (2013) [75] showed R^2^ = 0.9433. Dmowski and Post (2018) [49] in research on the impact of brewing times on the antioxidant activity (DPPH) of Yerba Mate, showed a significant correlation coefficient for both the first (R^2^ = 0.827) and the third brewing (R^2^ = 0.841). Other studies have also shown a significant correlation between antioxidant activity and the content of selected phytonutrients. Bassani et al. (2013) [48] showed a relationship between the content of flavonoids and the antioxidant activity measured with the use of DPPH radicals (R^2^ = 0.9046 to R^2^ = 0.9972). Deladino et al. (2013) [75] found a correlation between chlorogenic acid vs. DPPH (R^2^ = 0.8429) and gallic acid vs. DPPH (R^2^ = 0.9807). Other authors have also confirmed that Yerba Mate has strong antioxidant properties, resulting, among others, from the high content of phenolic components [4,8,9,15,16,17,20,21,22,23,48,77].

## 3. Materials and Methods

### 3.1. Materials

The research material consisted of three market Yerba Mate products (from Paraguay), the most frequently chosen brand in Poland. The products were purchased in an online store from a legal distributor in Poland, all in 500 g individual packages. The products were selected to represent the different types of Yerba Mate, i.e., “despalada sin palo” (containing up to 10% twigs and sticks) and “elaborada con palo” (containing up to 30% twigs and sticks), where, according to the manufacturer’s, there were 3 types of Yerba Mate, i.e., “Premium Despaada” (sample YM-A), “Elaborada Selección Especial” (sample YM-B) and “Elaborada Con Palo Tradicional” (sample YM-C). The research was carried out in dried samples and in Yerba Mate infusions.

The infusions were prepared at 70 °C and 100 °C, as a result of a single and double brewing process. To prepare the infusions, 5.0 g of dry samples were weighed into 100 mL glass beakers, poured over with 100 mL of distilled water at 70 °C and 100 °C, brewed for 10 min under cover, and then filtered (single brewing). The brewing process was repeated in an analogous way on the residues filtered from the first brewing (double brewing). The clear infusions obtained in this way were used for research.

### 3.2. Methods

The tests were carried out on dried samples (organoleptic evaluation, instrumental measurement of color, water activity, dry matter and moisture content) and Yerba Mate infusions (organoleptic evaluation, instrumental measurement of color, pH, osmolality, soluble solids content (°Brix), tannins, total polyphenols content and antioxidant activity).

#### 3.2.1. Dry Matter (d.m.), Moisture Content (%) and Water Activity (*a_w_*) in Dried Yerba Mate

The dry matter content (d.m.) was determined gravimetrically according to AOAC (2000) [78]. Yerba Mate samples (5.0 g) were weighed into weighing bottles on an analytical balance (AS 220/X, Radwag, Radom, Poland) and dried (105 °C, 72 h) in a laboratory dryer (SUP 200W, Wamed, Warsaw, Poland). The dry matter content and moisture content were calculated from the weight differences and the results are expressed as percentage (%).

Water activity (a_w_) was measured using a handheld AquaLab Water Activity Meter (Decagon Devices. Inc., Pullman, WA, USA) with a temperature stabilizer.

Measurements of dry matter content (d.m.), moisture content (%) and water activity (a_w_) were performed in three independent repetitions.

#### 3.2.2. Color Parameters in the CIE LAB (L*a*b*) Color Space and Color Functions in Yerba Mate

Color parameters of dried Yerba Mate and its infusions were measured at room temperature (20 °C) using a colorimeter (Konica Minolta CR-400, Konica Minolta, BSP, Warsaw, Poland) in the CIE Lab color space (L*a*b*) (*L*-brightness; +*a*-red; −*a*-green; +*b*-yellow; −*b*-blue). After calibration using a white CR-A43 reflective plate and placing the samples in a glass dish (Ø 60 mm) on the measuring head, the results were read.

Measurements of color parameters of dried Yerba Mate and its infusions were performed in three independent repetitions.

On the basis of the obtained chromatic coordinates L*a*b*, color functions, i.e., browning index (*BI*) and total color difference (Δ*E*) were calculated.

According to Maskan (2001) [54], the browning index (BI) was calculated using the following equations:

(1) $$BI=\frac{\left[100\left(x-0.31\right)\right]}{0.17}$$

where

(2) $$x=\frac{\left(a*+1.75L*\right)}{\left(5.654L*+a*-3.012b*\right)}$$

According to Schmalko et al. (2014) [47], the total color difference (Δ*E*) in Yerba Mate infusions were calculated using the following equation:

(3) $$\Delta E={\left[{\left(L-L_{0}\right)}^{2}+{\left(a-a_{0}\right)}^{2}+{\left(b-b_{0}\right)}^{2}\right]}^{\frac{1}{2}}$$

where: *L*_0_, *a*_0_, *b*_0_—the values obtained for dry Yerba Mate samples (before brewing)

#### 3.2.3. pH, Soluble Solids Content (°Brix) and Osmolality in Yerba Mate Infusions

The pH was measured by the potentiometric method using a laboratory pH-meter probe (Elmetron CP-511, Elmetron G.P., Zabrze, Poland), at room temperature (20 °C).

The soluble solids content (°Brix) was measured using an Abbe refractometer (ORT-1, Kern & Sohn GmbH, Balingren-Frommern, Germany), using the refractometric method, according to the Polish Standard (PN-EN 12143:2000) [79]. The results (expressed in %) were read from the sugar scale at room temperature (20 °C).

The osmolality of Yerba Mate infusions was measured using an osmometer (Osmometr Krioskop 800CL, Trident Med, Warsaw, Poland) by measuring the crystallization temperature of a supercooled solution with a one-point calibration. After measuring 100.0 μL of the infusion into the osmometric tube and placing it in the cooling chamber, as a result of supercooling the sample and initiating crystallization, the heat of crystallization is measured with a thermistor and automatically converted to mOsm/kg•H_2_O.

Measurements of pH, soluble solids content (°Brix) and osmolality in Yerba Mate infusions were performed in three independent repetitions.

#### 3.2.4. Tannin Content in Yerba Mate

The tannin content in the Yerba Mate infusions was determined using the titration and weight method according to Ciszewska et al. (1975) [80]. This method is based on the precipitation of proteins, and, after adding the 10% potassium iodide, titration of the secreted tannins with 0.05 M sodium thiosulfate solution in the presence of starch.

To determine the tannin content, 175 mL of Yerba Mate infusion was used, which was heated to boiling, 20 mL of a 4% solution of copper (II) acetate (CuSO_4_) (Sigma-Aldrich, Poznań, Poland) was added, then quantitatively transferred to a volumetric flask with a capacity of 200 mL, and after cooling, made up to the mark with distilled water and filtered through a pleated filter. Into a conical flask, 100 mL of filtrate was measured, 25 mL of 50% acetic acid (Sigma-Aldrich, Poznań, Poland), 20 mL of 10% potassium iodide (KI) solution and 1.5 mL of 2% aqueous starch solution (indicator) were added and titrated with sodium thiosulfate solution (0.05 M Na_2_S_2_O_3_) (Sigma-Aldrich, Poznań, Poland). The tannin content was expressed as g/100 g d.m. (d.m.—dry matter). The determination of tannin content was performed in three independent repetitions.

#### 3.2.5. Selectwed Bioactive Compounds Identified by HPLC in Yerba Mate

##### Carotenoids in Yerba Mate

Carotenoids content in Yerba Mate infusions was determined using the HPLC method according to Hallmann and Sabała (2020) [81]. Yerba Mate samples were extracted with cold acetone, then magnesium carbonate (MgCO_3_) was added and incubated in a cold ultrasonic bath (35 kHz, 0 °C, 15 min) (Bandelin Sonorex RK 255, BANDELIN Electronic, Berlin, Germany). After extraction, the samples were centrifuged (5500 rpm, 2 °C, 10 min) (MPW-380 R, MPW Med. Instruments, Poland, Warsaw). For the next step of analysis, 1 mL of centrifuged extracts were transferred into HPLC vials and used for HPLC analysis. The analysis was performed using equipment from Shimadzu (USA Manufacturing Inc., New Providence, IA, USA): two LC-20AD pumps, CMB-20A and CTD-20AC system controllers, SIL-20AC autosampler, CTD-20A oven and SPD-20AV UV/VIS detector (spectrophotometer). For this purpose, 50 μL of the supernatant was injected into a Max RP-80 A chromatographic column (250 × 4.6 mm, Phenomenex, Warsaw, Poland). Two mobile phases were used for the analytical procedure. The first mobile phase (A) contained 90% acetonitrile and 10% methanol. The second phase (B) contained 68% methanol and 32% ethyl acetate with a flow rate of 1 mL/min and a time program of 1.00–14.99 min, phase A 100%; 15.00–22.99 min, phase A 40% and phase B 60%; and 24.00–28.00 min, phase A 100%. The wavelength used for detection was 450–480 nm. Carotenoids were identified based on Fluka and Sigma Aldrich external standards (lutein, α-carotene and β-carotene) with a purity of 99.9%. All reagents were from Sigma-Aldrich (Sigma-Aldrich, Poznań, Poland). Carotenoids content was expressed as μg/g d.m. The determination of carotenoids content was performed in three independent repetitions.

##### Phenolic Acids, Flavonoids and Caffein in Yerba Mate

Individual phenolic acids, flavonoids and caffeine content in Yerba Mate infusions were determined using the HPLC method according to Hallmann and Sabała (2020) [81]. Briefly, 1 mL samples were extracted with 5 mL of 80% methanol in an ultrasonic bath (35 kHz, 30 °C, 15 min) (Bandelin Sonorex RK 255, BANDELIN Electronic, Berlin, Germany). After extraction, the samples were centrifuged (6000 rpm, 0 °C, 10 min) (MPW-380 R, MPW Med. Instruments, Poland, Warsaw) and 1 mL of centrifuged extracts prepared in this way were transferred into HPLC vials and used for HPLC analysis. The analysis was performed using equipment from Shimadzu (USA Manufacturing Inc., New Providence, IA, USA): two LC-20AD pumps, CMB-20A and CTD-20AC system controllers, SIL-20AC autosampler, CTD-20A oven and SPD-20AV UV/VIS detector (spectrophotometer). For this purpose, 50 μL of the supernatant was injected into a Fusion RP-80 A chromatographic column (250 × 4.6 mm, Phenomenex, Warsaw, Poland). Two mobile phases were used for analytical purposes. The first mobile phase (A) contained acetonitrile. The second phase (B) contained phosphoric acid water (pH 3.0) with a flow rate of 1 mL/min and a time program of 1.00–22.99 min, 95% phase A and 5% phase B; 23.00–27.99 min, 50% phase A and 50% phase B; 28.00–28.99 min, 80% phase A and 20% phase B; and 29.00–38.00 min, 95% phase A and 5% phase B. A single run lasted 42 min. The wavelength used for detection was 250–360 nm. Phenolic acids, flavonoids and caffeine were identified based on retention time of Sigma Aldrich external standards (gallic acid, chlorogenic acid, coffeic acid, p-coumaric acid, ferulic acid, salicylic acid, t-cinnamic acid, rutoside-3-O-quercetin, glycoside-3-O-quercetin, myricetin, apigenin and caffeine) with a purity of 99.9%. All reagents were from Sigma-Aldrich (Poznań, Poland). Phenolic acids, flavonoids and caffeine content was expressed as mg/g d.m. The determinations of individual phenolic acids, flavonoids and caffeine content were performed in three independent repetitions.

#### 3.2.6. Total Polyphenol Content in Yerba Mate

Total polyphenol content in Yerba Mate infusions was determined using the Folin-Ciocalteu reagent (Sigma-Aldrich, Poznań, Poland) according to Singleton and Rossi (1965) [82] method. The solution (1.0 mL of diluted Yerba Mate infusion, 2.5 mL of Folin-Ciocalteu reagent, 5.0 mL of 20% Na_2_CO_3_ (sodium carbonate) (Sigma-Aldrich, Poznań, Poland) in 41.5 mL of distilled water was incubated for 60 min (20 *°C*, no access to light) and the absorbance was measured in a spectrophotometer (UV/Vis UV-6100A, Metash Instruments Co., Ltd., Shanghai, China) at λ = 750 nm. After taking into account the dilutions used, the obtained results of absorbance measurements were recalculated based on the standard curve for gallic acid (Sigma-Aldrich, Poznań, Poland) as a standard substance and the total polyphenol content was expressed as mg GAE/g d.m. (GAE—gallic acid equivalent). The determination of total polyphenol content was performed in six independent repetitions.

#### 3.2.7. Antioxidant Activity in Yerba Mate

Antioxidant activity in Yerba Mate infusions was determined using the cation radical ABTS^+•^ (2,2′-azino-bis 3-ethylbenzothiazolin-6-sulfonic acid) (Sigma-Aldrich, Poznań, Poland) according to Re et al. (1999) [83] method. The solution (1.5 mL of diluted Yerba Mate infusion, 3.0 mL radical cations ABTS^+•^ in PBS solution (PBS—Phosphate Buffer Solution)) was vortexed (Wizard Advanced IR Vortex Mixer, VELP Scientifica Srl, Usmate (MB), Italy) and incubated for 6 min (20 °C) and the absorbance was measured in a spectrophotometer (UV/Vis UV-6100A, Metash Instruments Co., Ltd., Shanghai, China) at λ = 734 nm. After taking into account the dilutions used, the obtained results of absorbance measurements were recalculated based on the standard curve for Trolox (Sigma-Aldrich, Poznań, Poland) as a standard substance and the antioxidant activity was expressed as μM TEAC/g d.m. (TEAC—Trolox Equivalent Antioxidant Capacity). The determination of antioxidant activity was performed in six independent repetitions.

#### 3.2.8. Statistical Analysis

The results in tables and figures are presented as mean values ± standard deviation of 3–6 independent repetitions. One-way analysis of variance (ANOVA, Duncan’s post hoc test) was performed using the Statistica 13.0 software (Tibco Software Inc., Palo Alto, CA, USA). The differences between the samples were considered statistically significant at *p* ≤ 0.05.

## 4. Conclusions

Yerba Mate infusions, characterized by an intense flavor with herbal and smoky notes and a color similar to green, exhibit an osmolality of less than 100 mOsm/kg•H_2_O, so they belong to the hypotonic beverage group, just like mineralized waters, tea, or coffee. These infusions also show a low extract content and a pH close to neutral. After the second brewing, a reduction in the intensity of all the parameters listed above can be observed, while the brewing temperature had less of an effect on the less intense taste of the infusions. A more intense taste of Yerba Mate, especially bitter, is associated with a higher content of tannins in the infusions from the first brewing compared to the second; additionally, at higher temperatures the presence of more compounds belonging to this group in the infusion was noted compared to brewing at lower temperature. The opposite trend was noted for the content of phenolic compounds and antioxidant activity—the higher the brewing temperature and brewing multiplicity, the lower the content of bioactive ingredients. Therefore, in order to enjoy an infusion with the highest content of bioactive compounds and exhibiting a more intense flavor and color, Yerba Mate should be brewed at a lower temperature and drunk after the first brewing. However, if a less intense flavor infusion is needed, drinking it after the second brewing is also valuable due to the still high concentration of caffeine, polyphenolic compounds, carotenoids, tannins, and antioxidant activity.

## Figures and Tables

**Table 1:** Moisture content (%), water activity (*a_w_*), chromatic coordinates in the CIE LAB color space (L*a*b*) and browning index (BI) of dried Yerba Mate samples.

Sample	Moisture (%)	Water Activity (*a_w_*)	L* (*Lightness*)	a* (*Greenness*)	b* (*Yellowness*)	BI (*Browning Index*)
YM-A	5.58 ± 0.04 ^b^	0.38 ± 0.00 ^b^	48.78 ± 5.04 ^b^	−0.58 ± 0.17 ^c^	34.87 ± 3.48 ^b^	110.93 ± 6.10 ^b^
YM-B	5.86 ± 0.04 ^c^	0.37 ± 0.00 ^a^	37.63 ± 1.63 ^a^	−1.45 ± 0.14 ^b^	27.95 ± 1.30 ^a^	115.64 ± 2.12 ^b^
YM-C	5.05 ± 0.02 ^a^	0.38 ± 0.00 ^b^	74.00 ± 3.31 ^c^	−2.25 ± 0.38 ^a^	32.52 ± 1.84 ^b^	52.91 ± 2.28 ^a^

Mean values ± standard deviation with different letters (^a–c^) in the same column differ significantly (Duncan’s test*, p* ≤ 0.05).

**Table 2:** Chromatic coordinates in the CIE LAB (L*a*b*) color space, browning index (BI) and total color difference (Δ*E*) of Yerba Mate infusions obtained as a result of single brewing at 70 °C and 100 °C.

Sample	L* (*Lightness*)	a* (*Redness*)	b* (*Yellowness*)	BI (*Browning Index*)	Δ*E*
YM-A-1-70	32.30 ± 0.10 ^b^	12.10 ± 0.40 ^b^	39.95 ± 0.15 ^b^	360.26 ± 0.31 ^e^	21.41 ± 0.28 ^b^
YM-B-1-70	43.80 ± 1.70 ^e^	9.35 ± 0.15 ^a^	45.10 ± 0.60 ^e^	235.74 ± 12.72 ^a^	21.22 ± 0.90 ^ab^
YM-C-1-70	38.30 ± 0.40 ^d^	15.40 ± 0.30 ^de^	44.75 ± 0.35 ^e^	316.68 ± 2.24 ^d^	41.66 ± 0.37 ^d^
YM-A-1-100	30.60 ± 0.20 ^a^	15.72 ± 0.19 ^e^	34.05 ± 0.25 ^a^	290.60 ± 8.19 ^b^	24.43 ± 0.26 ^c^
YM-B-1-100	36.15 ± 0.65 ^c^	14.68 ± 0.63 ^cd^	40.75 ± 0.05 ^c^	293.85 ± 10.22 ^bc^	20.66 ± 0.48 ^a^
YM-C-1-100	37.60 ± 0.30 ^d^	14.18 ± 0.60 ^c^	43.45 ± 0.25 ^d^	308.39 ± 9.45 ^cd^	41.41 ± 0.44 ^d^

Mean values ± standard deviation with different letters (^a–e^) in the same column differ significantly (Duncan’s test, *p* ≤ 0.05).

**Table 3:** Color parameters in the CIE LAB (L*a*b*) color space, browning index (BI) and total color difference (Δ*E*) of Yerba Mate infusions obtained as a result of double brewing at 70 °C and 100 °C.

Sample	L* (*Lightness*)	a* (*Redness*)	b* (*Yellowness*)	BI (*Browning Index*)	Δ*E*
YM-A-2-70	60.00 ± 0.20 ^c^	−1.88 ± 0.13 ^c^	43.60 ± 0.40 ^b^	112.06 ± 0.93 ^b^	14.28 ± 0.41 ^a^
YM-B-2-70	57.30 ± 1.60 ^b^	−2.55 ± 0.15 ^b^	39.15 ± 0.35 ^a^	100.32 ± 5.72 ^a^	22.68 ± 1,21 ^d^
YM-C-2-70	55.05 ± 0.25 ^a^	−1.38 ± 0.13 ^d^	45.10 ± 0.70 ^c^	138.62 ± 2.57 ^c^	22.77 ± 0.17 ^d^
YM-A-2-100	57.15 ± 0.35 ^b^	−0.58 ± 0.08 ^e^	49.15 ± 0.35 ^d^	152.87 ± 3.65 ^d^	16.56 ± 0.12 ^b^
YM-B-2-100	57.65 ± 0.85 ^b^	−0.70 ± 0.05 ^e^	48.65 ± 0.15 ^d^	147.45 ± 3.07 ^d^	28.81 ± 0.70 ^e^
YM-C-2-100	58.15 ± 0.65 ^b^	−2.85 ± 0.05 ^a^	39.85 ± 0.40 ^a^	100.23 ± 0.21 ^a^	17.48 ± 0.42 ^c^

Mean values ± standard deviation with different letters (^a–e^) in the same column differ significantly (Duncan’s test, *p* ≤ 0.05).

**Table 4:** Physicochemical properties of Yerba Mate infusions obtained as a result of single brewing at 70 °C and 100 °C.

Sample	pH	°Brix (%)	Osmolality (mOsm/kg•H_2_O)
YM-A-1-70	5.56 ± 0.03 ^d^	2.07 ± 0.06 ^ab^	47.33 ± 0.58 ^b^
YM-B-1-70	5.41 ± 0.01 ^b^	2.07 ± 0.06 ^ab^	46.33 ± 0.58 ^ab^
YM-C-1-70	5.39 ± 0.01 ^ab^	2.07 ± 0.06 ^ab^	45.33 ± 0.58 ^a^
YM-A-1-100	5.52 ± 0.01 ^c^	2.17 ± 0.06 ^b^	55.33 ± 0.58 ^d^
YM-B-1-100	5.38 ± 0.00 ^a^	2.07 ± 0.06 ^ab^	51.00 ± 1.00 ^c^
YM-C-1-100	5.39 ± 0.00 ^ab^	2.03 ± 0.06 ^a^	51.33 ± 0.58 ^c^

Mean values ± standard deviation with different letters (^a–d^) in the same column differ significantly (Duncan’s test, *p* ≤ 0.05).

**Table 5:** Physicochemical properties of Yerba Mate infusions obtained as a result of double brewing at 70 °C and 100 °C.

Sample	pH	°Brix (%)	Osmolality (mOsm/kg•H_2_O)
YM-A-2-70	5.72 ± 0.01 ^e^	0.83 ± 0.15 ^c^	18.33 ± 0.58 ^d^
YM-B-2-70	5.54 ± 0.01 ^c^	0.60 ± 0.00 ^a^	12.33 ± 0.58 ^b^
YM-C-2-70	5.48 ± 0.00 ^a^	0.70 ± 0.00 ^ab^	11.67 ± 0.58 ^a^
YM-A-2-100	5.61 ± 0.02 ^d^	0.77 ± 0.06 ^bc^	14.67 ± 0.58 ^c^
YM-B-2-100	5.51 ± 0.01 ^b^	0.70 ± 0.00 ^ab^	14.33 ± 0.58 ^c^
YM-C-2-100	5.50 ± 0.00 ^b^	0.70 ± 0.00 ^ab^	12.67 ± 0.58 ^b^

Mean values ± standard deviation with different letters (^a–e^) in the same column differ significantly (Duncan’s test, *p* ≤ 0.05).

**Table 6:** Selected phenolic compounds identified by the HPLC method in the tested Yerba Mate infusions obtained as a result of single brewing at 70 °C and 100 °C.

Bioactive Compounds (mg/g d.m.)	YM-A-1-70	YM-B-1-70	YM-C-1-70	YM-A-1-100	YM-B-1-100	YM-C-1-100
Gallic acid	0.45 ± 0.00 ^a^	2.25 ± 0.03 ^c^	2.45 ± 0.08 ^d^	0.47 ± 0.00 ^a^	0.55 ± 0.02 ^b^	2.40 ± 0.01 ^d^
Chlorogenic acid	25.64 ± 0.36 ^c^	24.47 ± 0.03 ^b^	27.05 ± 0.86 ^d^	24.15 ± 0.16 ^b^	7.94 ± 0.12 ^a^	25.30 ± 0.43 ^c^
Caffeic acid	3.82 ± 0.08 ^b^	35.86 ± 2.54 ^a^	0.62 ± 0.03 ^a^	3.68 ± 0.04 ^b^	23.11 ± 0.56 ^c^	0.43 ± 0.04 ^a^
*p*-coumaric acid	38.15 ± 0.04 ^e^	32.20 ± 0.39 ^b^	36.81 ± 0.14 ^d^	38.56 ± 0.29 ^e^	12.86 ± 0.86 ^a^	34.01 ± 0.44 ^c^
Ferulic acid	1.87 ± 0.02 ^c^	0.31 ± 0.02 ^a^	0.36 ± 0.00 ^a^	1.64 ± 0.02 ^b^	3.31 ± 0.09 ^d^	0.30 ± 0.01 ^a^
Salicylic acid	1.98 ± 0.02 ^d^	0.51 ± 0.06 ^a^	0.69 ± 0.02 ^b^	0.56 ± 0.01 ^a^	0.91 ± 0.05 ^c^	0.56 ± 0.00 ^a^
*t*-cinnamic acid	7.03 ± 0.08 ^c^	7.48 ± 0.03 ^e^	7.30 ± 0.07 ^d^	2.77 ± 0.01 ^a^	8.43 ± 0.09 ^f^	6.08 ± 0.05 ^b^
Total phenolic acids *	**78.95 ± 0.49 ^f^**	**67.58 ± 0.82 ^b^**	**75.29 ± 0.92 ^e^**	**71.83 ± 0.18 ^d^**	**57.10 ± 0.61 ^a^**	**69.09 ± 0.45 ^c^**
Rutoside-3-*O*-quercetin	2.60 ± 0.04 ^e^	2.11 ± 0.07 ^d^	2.02 ± 0.01 ^c^	1.18 ± 0.04 ^b^	0.93 ± 0.01 ^a^	0.93 ± 0.03 ^a^
Glycoside-3-*O*-quercetin	4.08 ± 0.07 ^e^	3.19 ± 0.03 ^c^	3.54 ± 0.03 ^d^	1.40 ± 0.07 ^a^	1.87 ± 0.02 ^b^	1.90 ± 0.03 ^b^
Myricetin	0.94 ± 0.01 ^d^	0.75 ± 0.01 ^b^	0.90 ± 0.01 ^c^	1.64 ± 0.02 ^e^	0.65 ± 0.01 ^a^	0.63 ± 0.00 ^a^
Apigenin	0.10 ± 0.01 ^b^	0.54 ± 0.02 ^c^	0.70 ± 0.01 ^d^	0.06 ± 0.00 ^a^	0.09 ± 0.00 ^ab^	0.09 ± 0.00 ^b^
Total flavonoids *	**7.71 ± 0.09 ^e^**	**6.59 ± 0.12 ^c^**	**7.16 ± 0.06 ^d^**	**4.28 ± 0.05 ^b^**	**3.54 ± 0.03 ^a^**	**3.56 ± 0.03 ^a^**
Lutein ^#^	8.49 ± 0.03 ^c^	8.02 ± 0.07 ^a^	8.19 ± 0.03 ^b^	8.40 ± 0.04 ^c^	7.99 ± 0.05 ^a^	8.27 ± 0.08 ^b^
α-carotene ^#^	3.50 ± 0.01 ^c^	3.37 ± 0.01 ^a^	3.46 ± 0.01 ^b^	3.48 ± 0.02 ^bc^	3.36 ± 0.01 ^a^	3.47 ± 0.01 ^b^
β-carotene ^#^	1.10 ± 0.05 ^b^	0.81 ± 0.05 ^a^	1.27 ± 0.08 ^bc^	1.70 ± 0.04 ^d^	1.32 ± 0.04 ^c^	1.19 ± 0.23 ^bc^
Total carotenoids *^#^	**13.09 ± 0.07 ^c^**	**12.20 ± 0.08 ^a^**	**12.92 ± 0.04 ^c^**	**13.58 ± 0.08 ^d^**	**12.67 ± 0.08 ^b^**	**12.93 ± 0.28 ^c^**

Mean values ± standard deviation with different letters (^a–f^) in the same line differ significantly (Duncan’s test, *p* ≤ 0.05). *—total phenolic acids, total flavonoids and total carotenoids expressed as the sum of identified bioactive compounds. ^#^—lutein, α-carotene, β-carotene, total carotenoids expressed in μg/g d.m.

**Table 7:** Selected phenolic compounds identified by the HPLC method in the tested Yerba Mate infusions obtained as a result of double brewing at 70 °C and 100 °C.

Bioactive Compounds (mg/g d.m.)	YM-A-2-70	YM-B-2-70	YM-C-2-70	YM-A-2-100	YM-B-2-100	YM-C-2-100
Gallic acid	2.44 ± 0.09 ^e^	0.43 ± 0.00 ^a^	0.89 ± 0.02 ^b^	0.87 ± 0.01 ^b^	1.23 ± 0.04 ^c^	1.46 ± 0.09 ^d^
Chlorogenic acid	3.28 ± 0.11 ^a^	15.24 ± 0.45 ^e^	5.47 ± 0.07 ^b^	7.89 ± 0.08 ^d^	6.64 ± 0.04 ^c^	5.62 ± 0.13 ^b^
Caffeic acid	30.90 ± 0.22 ^f^	12.81 ± 0.07 ^c^	12.21 ± 0.34 ^b^	22.63 ± 0.07 ^d^	2.41 ± 0.02 ^a^	24.40 ± 0.14 ^e^
*p*-coumaric acid	8.93 ± 0.10 ^a^	19.92 ± 0.12 ^d^	39.80 ± 0.20 ^e^	14.67 ± 0.16 ^b^	16.92 ± 0.11 ^c^	8.87 ± 0.10 ^a^
Ferulic acid	4.25 ± 0.49 ^c^	5.33 ± 0.06 ^d^	3.67 ± 0.05 ^b^	3.23 ± 0.01 ^a^	4.14 ± 0.06 ^c^	5.33 ± 0.02 ^d^
Salicylic acid	2.62 ± 0.13 ^d^	1.57 ± 0.05 ^c^	0.48 ± 0.00 ^a^	0.95 ± 0.04 ^b^	1.47 ± 0.03 ^c^	3.08 ± 0.01 ^e^
*t*-cinnamic acid	13.50 ± 0.29 ^f^	1.73 ± 0.04 ^a^	2.71 ± 0.01 ^b^	9.06 ± 0.05 ^d^	7.51 ± 0.25 ^c^	9.95 ± 0.05 ^e^
Total phenolic acids *	**65.91 ± 0.62 ^e^**	**57.03 ± 0.46 ^b^**	**65.23 ± 0.18 ^d^**	**59.31 ± 0.01 ^c^**	**40.32 ± 0.21 ^a^**	**58.71 ± 0.37 ^c^**
Rutoside-3-*O*-quercetin	1.25 ± 0.02 ^a^	1.60 ± 0.03 ^b^	2.01 ± 0.01 ^c^	1.58 ± 0.02 ^b^	1.55 ± 0.04 ^b^	1.58 ± 0.04 ^b^
Glycoside-3-*O*-quercetin	1.51 ± 0.02 ^d^	0.99 ± 0.01 ^b^	1.07 ± 0.01 ^c^	0.90 ± 0.02 ^a^	1.00 ± 0.03 ^b^	0.93 ± 0.03 ^a^
Myricetin	2.34 ± 0.00 ^f^	0.50 ± 0.01 ^b^	0.68 ± 0.00 ^d^	0.84 ± 0.01 ^e^	0.45 ± 0.01 ^a^	0.52 ± 0.01 ^c^
Apigenin	0.08 ± 0.00 ^b^	0.08 ± 0.00 ^ab^	0.13 ± 0.00 ^c^	0.07 ± 0.02 ^ab^	0.06 ± 0.00 ^a^	0.14 ± 0.01 ^c^
Total flavonoids *	**5.18 ± 0.03 ^e^**	**3.16 ± 0.04 ^b^**	**3.89 ± 0.00 ^d^**	**3.38 ± 0.03 ^c^**	**3.06 ± 0.03 ^a^**	**3.18 ± 0.04 ^b^**
Lutein ^#^	9.25 ± 0.05 ^cd^	8.11 ± 0.07 ^a^	8.60 ± 0.05 ^b^	9.49 ± 0.15 ^d^	8.20 ± 0.28 ^a^	9.11 ± 0.05 ^c^
α-carotene ^#^	3.23 ± 0.02 ^bc^	3.15 ± 0.01 ^a^	3.33 ± 0.01 ^d^	3.26 ± 0.01 ^c^	3.21 ± 0.03 ^b^	3.22 ± 0.02 ^b^
β-carotene ^#^	0.65 ± 0.00 ^c^	0.58 ± 0.01 ^b^	0.82 ± 0.02 ^e^	0.69 ± 0.03 ^d^	0.52 ± 0.03 ^a^	0.59 ± 0.03 ^b^
Total carotenoids *^#^	**13.14 ± 0.05 ^c^**	**11.84 ± 0.08 ^a^**	**12.75 ± 0.03 ^b^**	**13.44 ± 0.17 ^d^**	**11.93 ± 0.30 ^a^**	**12.93 ± 0.02 ^bc^**

Mean values ± standard deviation with different letters (^a–f^) in the same line differ significantly (Duncan’s test, *p* ≤ 0.05). *—total phenolic acids, total flavonoids and total carotenoids expressed as the sum of identified bioactive compounds. ^#^—lutein, α-carotene, β-carotene, total carotenoids expressed in μg/g d.m.

## Data Availability

The original contributions presented in the study are included in the article, further inquiries can be directed to the corresponding authors.
